# Development and validation of a natural dynamic facial expression stimulus set

**DOI:** 10.1371/journal.pone.0287049

**Published:** 2023-06-28

**Authors:** Laura Pasqualette, Sara Klinger, Louisa Kulke

**Affiliations:** 1 Neurocognitive Developmental Psychology, Psychology Department, Friedrich-Alexander Universität Erlangen-Nürnberg, Erlangen, Bavaria, Germany; 2 Developmental and Educational Psychology Department, University of Bremen, Bremen, Germany; Kuwait College of Science and Technology, KUWAIT

## Abstract

Emotion research commonly uses either controlled and standardised pictures or natural video stimuli to measure participants’ reactions to emotional content. Natural stimulus materials can be beneficial; however, certain measures such as neuroscientific methods, require temporally and visually controlled stimulus material. The current study aimed to create and validate video stimuli in which a model displays positive, neutral and negative expressions. These stimuli were kept as natural as possible while editing timing and visual features to make them suitable for neuroscientific research (e.g. EEG). The stimuli were successfully controlled regarding their features and the validation studies show that participants reliably classify the displayed expression correctly and perceive it as genuine. In conclusion, we present a motion stimulus set that is perceived as natural and that is suitable for neuroscientific research, as well as a pipeline describing successful editing methods for controlling natural stimuli.

## Introduction

In social communication, the face plays a significant role [1], as it is, an important source of emotional and social information [2, 3]. To conduct research on emotion recognition and emotion perception, emotional facial expression stimuli are being used. In the past, these stimuli have consisted of static photographs of the peak of an emotional expression (e.g., [1]; the Pictures of Facial Affect, [4]; the Radbound Faces Database, [5], the McLellan Faces, [6]). However, dynamic video stimuli of an enfolding emotional expression have a higher ecological validity than static stimuli [3, 7–9]. Consequently, the use of dynamic stimuli has become more common recently (e.g., Bek et al., 2020 and Zinchenko et al., 2018 [10, 11]). Some dynamic stimuli databases have been created by using computer morphing software, which has proven useful, since temporal aspects and intensity levels of facial expression are easily adjustable with the software (e.g., Biele & Grabowska, 2006, Holland et al., 2019, Kamachi et al., 2013, Krumhuber & Kappas, 2005, Montagne et al., 2007, Recio et al., 2011, 2014 and Sato & Yoshikawa, 2004 [1, 12–18]). However, using computer morphing software might create unnatural facial expressions [18, 19]. Therefore, we aimed to create a natural dynamic facial expression stimulus set by recording real facial expressions of a model. To achieve stimuli that are perceived as genuine, two different elicitation methods (posed, event-elicited) were used to create positive, neutral and negative facial expressions. Posed expressions refer to expressions that are shown in the absence of an underlying emotional state [20]. Event-elicited emotional facial expressions are defined as reflecting genuine emotion, elicited by real, remembered or imagined events [21]. Common examples for event-elicited expressions are, a frightened expression due to being afraid of a present spider, or a sad expression caused by remembering the loss of a loved one [21]. Garrido et al. (2016) also made use of instructions to imagine specific situations that successfully elicited the respective emotion to create genuine facial expressions [22]. They also found that smiling expressions were perceived as most genuine compared to the other facial expressions (neutral and frowning expression) [22].

In the current study, three facial expressions (negative, neutral, and positive) were recorded each with the two elicitation methods (posed, event-elicited) under standardized conditions (lightning, background, sitting position, clothing, face-to-video ratio). After recording the dynamic facial expressions, they were standardized in video length, dynamic aspects (onset and peak expression duration), blinking amount, and luminance. Apart from the stimulus development, two validation studies of the developed stimuli are presented here. In these two studies, the stimuli were rated regarding valence, certainty and intensity at the emotion onset (study 1) and valence, intensity, genuineness and perceived edits for the full video (study 2). Valence is a qualitative component of emotions, which ranges from negative to positive [23, 24]. According to Sonnemans and Fridja (1994) intensity refers to an estimate of magnitude of the subjective impact of an emotional event or stimulus and it is probably one of the most noticeable aspects of an emotion [25]. As stated by Livingstone, Choi and Russo (2014) genuineness of emotional expression refers to the extent to which a given expression is considered a truthful reflection of a displayer’s physiological, mental or emotional state [26]. To validate facial expression databases, researchers have employed either highly trained raters i (e.g., Ekman & Friesen, 1977, 1978 [27, 28]). or untrained volunteers (e.g., CAFÉ, LoBue & Thrasher, 2015 [29]; NimStim, Tottenham et al., 2009 [30]; SAVE database, Garrdio et al., 2016 [22]). According to Tottenham et al. (2009) the use of untrained volunteers enhances ecological validity, as they may behavesimilarly to the samples that are recruited for studies using the developed databases [30]. Therefore, we chose to validate the stimuli on untrained participants in the current studies. Additionally, the underlying emotions of the confederate displaying the facial expressions and the mood of the observers in the two validation studies were measured.

The current project aimed (1) to develop emotional stimulus videos that are natural but sufficiently controlled to be used in laboratory research involving electroencephalography (EEG) or eye-tracking, (2) to validate these stimuli by using ratings of valence, intensity, genuineness and perceived edits [21], and (3) to investigate whether the elicitation method of emotional expressions changes ratings of perceived valence, intensity and genuineness. To allow for a use in neuroscientific research, the created stimulus set needs to be as standardized as possible [31], and controlled for luminance [32, 33].

### Valence perception of the different facial expressions

We expected positive and negative emotional expressions to be accurately recognized, due to typically high recognition rates for happy and angry expressions (e.g., Goeleven et al., 2008 and Langner et al., 2010 [5, 31]). Therefore, (H1) valence of video clips displaying a negative expression should be rated significantly negative, and (H2) valence of video clips displaying a positive expression should be rated significantly positive. While neutral expressions can have lower recognition rates than happy and angry expressions, they are mostly confused with other low valence emotions like anticipation or contempt [34]. Thus, we expected neutral expressions to still be rated with a low valence rating (i.e., a neutral rating), even if emotion labelling would possibly not be accurate. In other words, (H3) valence ratings of neutral videos should lie between negative and positive videos, significantly differing from both.

### Genuineness and intensity perception of the different facial expressions

Dawel et al. (2017) compared normative genuineness of facial expression stimuli of three databases, the Pictures of Facial Affect (PoFA, [4]), the Radboud Faces Database [5] and the McLellan Faces [6]. While facial expression stimuli that depicted a happy expression were rated as genuine in each of the three databases, the genuineness ratings of the negative expression stimuli (i.e., anger, disgust, fear and sadness) differed between the databases. Whereas the angry, disgusted, and fearful expressions of the PoFA and all negative expressions of the RaFD were perceived as fake, only sadness of the McLellan Faces was perceived as fake [21]. In conclusion, the perceived genuineness of negative expressions is more ambiguous, whereas happy expressions are generally perceived as genuine. Therefore, (H4) we expected that genuineness ratings of video clips displaying a negative expression vary more than genuineness ratings of the video clips displaying a neutral or a positive expression.

Biele and Grabowska (2006) investigated the perceived emotion intensity of static and dynamic facial expressions by presenting either pictures from the Montreal Set of Facial Expression of Emotion (MSFDE, [35]) or dynamic stimuli which were computer generated from the MSFDE pictures [1]. The MSFDE pictures display emotions at different intensities (neutral, 20, 40, 60 and 80% and full emotion display). These different pictures were morphed together to create the dynamic stimuli. The study of Biele and Grabowska (2006) revealed not only that dynamic expressions are perceived as more intense than static ones, but also, that angry expressions are rated as more intense than happy expressions. This could be due to individuals tending to overestimate the intensity of negative emotions [1], because negative emotions (e.g. anger) signal a possible threat [36]. Therefore, (H5) we expected that the intensity of the videos displaying a negative expression is rated significantly higher than the intensity of the videos displaying a positive expression.

In real-life social interactions, spontaneously displayed facial expressions are mostly of low to moderate intensity [37], with full intensity expressions being the exception [38]. With spontaneous subtle expressions commonly displayed as well as seen, they might be perceived as more genuine due to the model being able to show the expressions in a more typical way. Therefore, (H6) we expected the video clips rated as low to moderate intensity (0–4) are considered more genuine than the video clips rated as moderate to high intensity (5–9). Considering that variations of intensity levels of emotional expressions are not commonly included in facial expression stimulus sets [7], there is, to our knowledge, no research on the influence of varying intensity levels of facial expressions on genuineness perception.

### Influence of elicitation method of facial expression on genuineness perception

In the past, posed happy expressions have received relatively high ratings of genuineness (RaFD, [5]), sometimes even the highest ratings of genuineness compared to other emotional expressions [39, 40]. At the same time Dawel et al. (2017) reported ratings of perceived genuineness that differed significantly from zero only for event-elicited positive expressions, and not for posed ones (e.g., the happy expressions of the RaFD) [21]. This difference in genuineness ratings of posed expressions of the RaFD, once validated by Lagner et al. (2010), and once by Dawel et al. (2017), could be due to the optimized genuineness scale used by Dawel et al. (2017). However, the posed expressions of the RaFD, as validated by Dawel et al. (2017), were still not perceived as fake. Furthermore, Dawel et al. (2017) also reported multiple cases of stimuli that were posed and yet perceived as reflecting genuine emotions, in particular for happy expressions [21]. In fact, posed happy expressions were rarely perceived as truly fake with most of them being perceived as ambiguous or even as showing genuine emotion [21]. One possible reason for this lack of a difference in genuineness ratings for posed and event-elicited happy expressions is that displayers might be more experienced in posing a happy expression because of greater practice in everyday life [21]. In addition, possible differences in genuineness ratings for posed and event-elicited stimuli must be considered. In part, it could be that the temporal dynamics of positive expressions, which provide useful information for the judgement of genuine smiles [13, 41, 42]. Several studies have in fact shown that dynamic properties differ between genuine and fake (i.e., posed) smiles [13, 14, 20, 41–48]. As a result, of the standardization of the stimulus set, certain dynamic aspects (i.e., onset, and peak duration) had to be controlled, reducing the difference between the two elicitation conditions. Consequently, (H7) we did not expect to find a significant difference in genuineness ratings between the posed and the event-elicited video clips that are displaying positive expressions. The negative expression stimuli of the PoFA and the RaFD, which were all posed expressions, were all rated as fake in Dawel et al. (2017), whereas the normative ratings of the McLellan Faces did not reveal a clear difference between event-elicited and posed expressions for most negative expressions (i.e., anger, disgust, fear) [21]. Considering that posed negative expressions were generally perceived as fake and event-elicited negative expressions were not, we expect to find a difference in perceived genuineness between the posed and the event-elicited negative stimuli. To our knowledge, the little research on the effects of dynamic aspects on genuineness judgements has focused on positive expressions, while there is a lack of research on negative expressions. Due to the absence of evidence that the temporal dynamics of negative expressions play a similar role for the perception of genuineness as they do for positive expressions, we did not assume that the difference in genuineness between event-elicited and posed expressions is being reduced by the temporal standardization of the stimuli. Consequently, (H8) we expected that the event-elicited video clips displaying a negative expression are rated as more genuine than the posed video clips displaying a negative expression.

Finally, neutral facial expressions are seldom included in facial expression databases and were not explicitly validated by Dawel et al. (2017) [21]. Additionally, while dynamic aspects, like the speed of onset, may differ between elicitation conditions for positive and negative expression, there are close to no dynamic aspects within a neutral expression that could differ between the posed and event-elicited condition. Furthermore, for both elicitation conditions, the blinking times have been controlled to occur either before the first peak frame or after. Therefore, (H9) we expected that there is no difference in genuineness ratings between posed and event-elicited neutral expression video clips.

## Materials and methods—Development of natural dynamic facial expression stimuli

### Model

The facial expression stimuli were created by filming one person multiple times. The confederate (female, 28 years old, white Latin) had dark brown, short hair, brown eyes, wore a light grey pullover, and a light natural make-up consisting of mascara, concealer, and a lightly red tinted lip balm. Her hairstyle had little variances in the videos, which occurred naturally, if the changes were too drastic, the hairstyle was corrected by the confederate. The individual in this manuscript has given written informed consent (as outlined in PLOS consent form) to publish these case details.

### Materials and measurement

With a Logitech C920 Pro HD Webcam and the software Cheese (version 3.14.2, The GNOME Project) the dynamic stimuli were recorded. For video cutting the software DaVinci Resolve (version 17.4.2, Blackmagic Design) was used. The current mood of the confederate during the video recording was measured with the English Version of the PANAS [49] as was the case in Garrido et al. (2016) [21]. Demographic questions were asked before the experiment and five open questions regarding the success of the elicitation conditions were asked after the experiment in a written format (S1 File).

### Stimulus material

**Fig 1:**
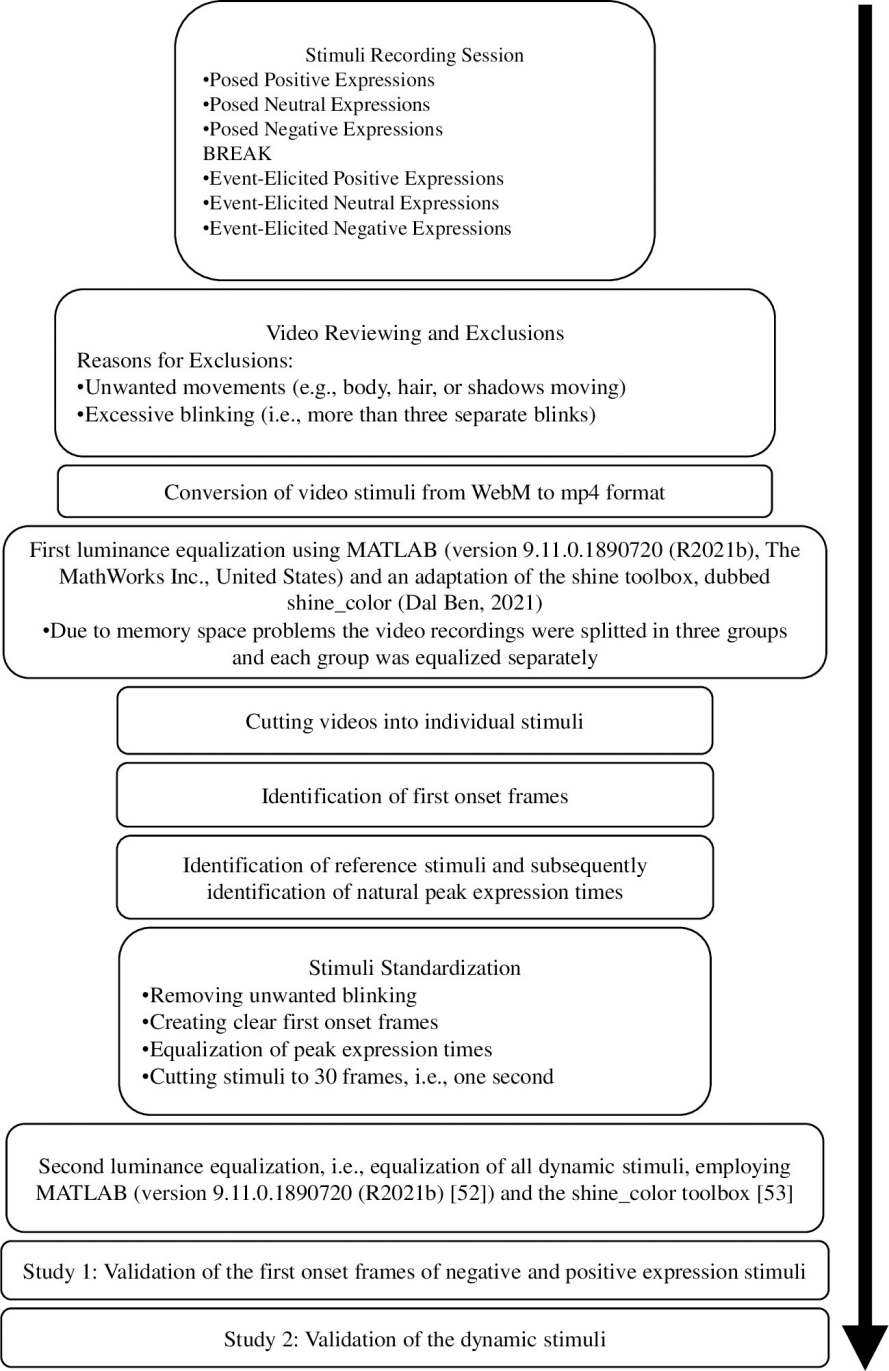
Development and validation process of the stimulus set.

**Fig 2:**
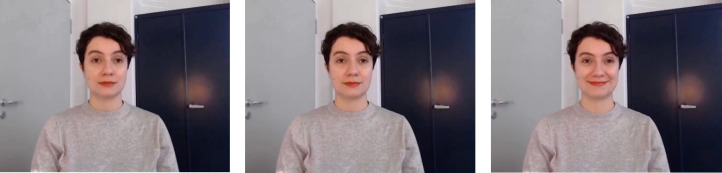
Typical Developing Positive Expression Stimuli from Neutral (Left) over First Onset Frame (Middle) to Peak Frame (Right).

**Fig 3:**
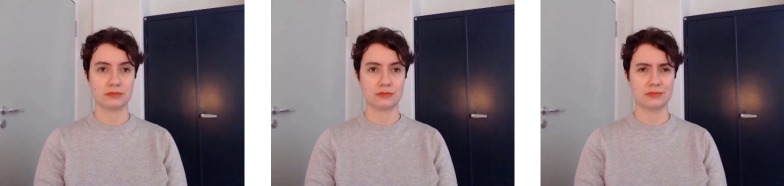
Typical Developing Negative Expression Stimuli from Neutral (Left) over First Onset Frame (Middle) to Peak Frame (Right).

An overview of the main steps taken in development and validation process can be seen in Fig 1. Dynamic stimuli were created in a deliberately posed condition and an event-elicited condition. In each condition positive, neutral, and negative expressions were displayed by the model. Instructions were prepared for both conditions and each expression. The model was instructed to show a stereotypical expression (positive, neutral, negative) in the posed- condition. In the event-elicited, instructed condition she was instructed to imagine a situation where she is to give a participant visual feedback (positive, no feedback, negative) on their performance in a study. The model knew about the two different elicitation conditions from the start, but was not aware of the exact instructions. In each condition three long videos were meant to be recorded, one for each expression, and in each long video the confederate was supposed to show the respective expression several times (approx. 60 times), always beginning with a neutral facial expression. Due to the wandering sun, light spots could be seen in some parts of some long videos. Therefore, certain videos had to be stopped and retaken, prolonged or an additional video had to be recorded. As a result, nine videos, instead of six, were created in total. After the recording, the videos were reviewed by the main experimenter. Some expressions had to be excluded due to them not fitting the standardization criteria because of e.g., body movements, or appearing shadows. The long videos were then cut into approximately 60 short video clips for each expression and condition. Subsequently, they were standardized and cut into clips with a length of one second. The mouth was closed in all video clips. Video clips that did not fit the standardization after closer inspection were excluded, as well as video clips with excessive blinking. The video clips include a maximum of one blink. Clips were kept in 960 × 720 pixel resolution and transformed from a WebM-format to mp4-format. All clips were recorded in colour. Fig 2 shows an example of a developing positive expression and Fig 3 shows an example of a developing negative expression.

#### Standardization criteria

We used the following standardization criteria: For the positive and negative expressions, each video clip contains the evolution of one expression (positive or negative) from a neutral facial expression (presented for approx. 33,3ms) over the onset of the emotional expression (for approx. 600ms) to the peak of the expression (from approx. 667ms to 1000ms). For each video clip, the onset and the peak of the expression was determined before the standardization process by research assistants. They followed a guide defining the emotional onset as the moment when any individual facial actions appeared [50], and based on Fujimura and Umemura (2018), the peak of an expression was defined as the point at which facial muscular activities could no longer be visually observed [51]. Mean peak expression time before the standardization of positive expression stimuli was 25.39 (*SD* = 5.68), and for negative expression stimuli it was 19.67 (*SD* = 5.68). As for the neutral expression video clips, there is no onset and peak phase, however, to include a dynamic aspect in those stimuli, the confederate blinks once in each video clip (based on the stimuli by Fujimura and Umemura, 2018 [51]), keeping the video length constant. In all the dynamic stimuli, it was ensured that the eyes were open at the beginning and the end of each clip, so blinking may occur in the video clip, but not right at the start (first two frames) or right at the end (30th frame). Additionally, the video stimuli should portray the confederate in the same sitting position with her head being the same size. Both were checked before a new video was filmed. Unnecessary movements of the body or head should not take place, as well as no excessive blinking.

*Controlling luminance*. The video stimuli were controlled for luminance. Before cutting the long videos the luminance of the video footage was equalized using MATLAB (version 9.11.0.1890720 (R2021b) [52]) and an adaptation of the SHINE toolbox, dubbed SHINE_color [53]. Due to the size and number of stimuli exceeding the memory limit, we had to split the video footage into three equal parts. Each part consisted of 114 expressions, i.e., 19 expressions of each condition, due to 57 being the highest amount of usable expressions in the posed neutral condition which was the condition with the fewest usable expressions. Within the equalization process, the SHINE_color toolbox extracted each frame of each long video, calculated its luminance, and then equalized each frame to fit the chosen luminance. As a result, each frame of the three videos has approximately the same mean luminance and *SD* (*M* = 127.41, *SD* = 0.001). After all further standardization steps were finished, the final set of video clips for the validation studies were equalized again to achieve an even more similar luminance.

*Editing the stimuli*. DaVinci Resolve 17 (version 17.4.2, [54]) was used for cutting and editing the videos. At first, the three equalized long videos were cut into approximately two-second-long video clips using the frames as reference. As the videos were filmed with a frame rate of 30 fps, a 2-second video clip consisted of 60 frames. After creating the 2-second video clips, the goal was to standardize each video clip, and in the end cut them into 1-second video clips, i.e., to a 30-frame format. The standardization criteria were as explained above.

*Blinking*. As a first step of standardizing the stimuli, all of the 2-second video clips were assessed regarding their temporal aspects. All clips underwent the basic edits of matching the peak expression time. Some clips already fulfilled most standardization criteria before any edits were made (reference clips), while other clips required further edits of deleting unnecessary frames during the emotional onset or removing a blink directly before the emotional onset. To ensure sufficient numbers of video stimuli these edits were made. The reference clips were used as a reference for all edits since these stimuli resembled the most natural stimuli best. The mean amounts of removed and added frames are in Table 1 for the reference and the other video clips. All undertaken edits per video clip are in the S1 Table.

**Table 1:** Undertaken edits in amount of frames removed or added.

Condition	Clip Category	Frames Removed Overall			Frames Added Overall	Edits due to Blinking		Edits to Create Clear Onset		Edits to Match Peak Times	
						Removed	Added	Removed	Added	Removed	Added
Positive Overall			8.61 *(6*.*15)*	0.55 *(0*.*96)*		4.14 *(3*.*95)*	0	0.99 *(1*.*10)*	0	3.55 *(4*.*97)*	0.55 *(0*.*96)*
	Reference Clips		5.03 *(3*.*58)*	0.11 *(0*.*47)*		0	0	1.31 *(2*.*58)*	0	3.71 *(3*.*30)*	0.11 *(0*.*47)*
	Additional Clips		11.12 *(5*.*41)*	0.86 *(1*.*54)*		7.04 *(2*.*18)*	0	0.76 *(2*.*79)*	0	3.44 *(4*.*84)*	0.86 *(1*.*54)*
Negative Overall			6.40 *(5*.*83)*	2.41 *(3*.*02)*		4.28 *(4*.*33)*	0	1.02 *(2*.*84)*	0	1.07 *(1*.*98)*	2.41 *(3*.*03)*
	Reference Clips		1.26 *(3*.*17)*	2.74 *(2*.*55)*		0	0	0.66 *(2*.*24)*	0	0.60 *(1*.*77)*	2.74 (2.55)
	Additional Clips		9.09 *(5*.*04)*	2.24 *(3*.*26)*		6.52 *(3*.*72)*	0	1.21 *(3*.*10)*	0	1.31 *(2*.*05)*	2.24 *(3*.*26)*
Neutral Overall			0	0		0	0	0	0	0	0

Mean number of frames removed or added and standard deviations in brackets. Reference clips refer to the video clips that were used as reference for the appropriate peak time. Additional clips refer to video clips that were not used to calculate the natural mean peak time due to including some aspect that did not fit the standardization criteria from the beginning and was therefore edited later.

All neutral clips include blinking as a dynamic aspect, which starts either during the emotional onset (100ms-533ms), or after the peak expression (667ms or later). The amount of neutral clips with blinking during the onset period was matched to the amount of clips with blinking in the onset period in the positive and negative conditions. Before the validation studies there were 62 such clips overall in the positive and negative conditions. So 62 neutral clips (30 event- elicited and 32 posed) were created with blinking starting during the onset period by simply cutting around the start of the blinking. No edits were made for the neutral clips.

*Clear onset expression*. The onset of an expression was defined as the moment when any individual facial actions appeared [50]. However, while the second frame displayed the start of the onset, the respective expression could not always be told by the second frame, which was especially the case for the negative expression stimuli. Consequently, we reviewed all video clips, and decided to delete unclear frames during the early onset to create a relatively clear and visible onset at the second frame for all positive and negative clips. The goal of these edits was to create standardized stimuli which would still be and appear as natural as possible. For the average edits per category see Table 1. All editing steps that were undertaken per stimulus can be found in the S1 Table.

*Matching peak expression times*. As mentioned above, the video clips that fitted most standardization criteria from the beginning (i.e., reference clips) were chosen as reference for all other clips, as those clips resembled the final stimuli best (i.e., no blinking at the start of the expression). One research assistant noted the peak times of these video clips. Afterwards, the most extreme values (highest and lowest 10% of each condition) were checked by the main experimenter and if necessary rectified. Additionally, three randomly chosen values were reviewed by the main experimenter as well. On average, the peak of the reference video clips was reached at the 20^th^ frame (approx. 667ms). Therefore, the 20^th^ frame was chosen as the frame which should result as peak after editing all video clips. To match peak times, frames were deleted during the onset period for video clips with the peak expression after the 20^th^ frame, which was the case for most of the positive video clips. Frames were added during the onset period for video clips in which the peak expression was reached before the 20^th^ frame, which was the case for most negative video clips. Often, a visible change in the facial expression appeared in every other frame, this observation was used to edit the video clips. If possible, only frames were deleted where no change was visible compared to the earlier frame. Frames were added by copying frames of the same clip and pasting them right after the copied frame. When possible, frames were copied and pasted if a frame with a visible change followed the respective frame, so there were not three frames in a row without a visible change.

### Procedure

The webcam was set up on a tripod in front of a computer screen. The middle of the webcam was placed at a height of 33cm, with the tripod being 30,5cm high. The confederate sat on a chair (armchair height: 61cm) facing the computer screen. The confederate’s nose was 64cm away from the webcam. Her gaze was directed at the computer screen where she could see herself displayed, so the videos would resemble a realistic video chat. The background was kept as neutral as possible, only a light grey door on the left, a white wall in the middle, and a dark blue locker on the right was displayed (natural laboratory environment). The head of the confederate was surrounded by the white wall to ensure the same contrast on both hemifaces. The shutters were closed for standardization reasons. Since the overhead lights caused flickering in the videos, they were turned off and a small table lamp was used instead. It was placed behind the computer screen, facing the wall, so no harsh shadows were created. Before each video, the distances and the face-to-video ratio were checked by measuring the confederate’s head size in the video while keeping the screen video the same size. After the preparations, the experimenter noted the starting time and handed out the first PANAS [49] to measure possible changes in the confederate’s mood before each video was recorded. In total nine long videos were created and 11 PANAS questionnaires were filled out by the confederate.

## Materials and methods—Study 1: First onset frame validation

### Participants

Fourteen participants between age 18 and 35 years (recruited e.g., via institutional email, social contacts) voluntarily took part in the online survey (9 female, 4 male) after giving written informed consent. One of the participants was excluded from all calculations due to not finishing the study. The participants included in the analyses were between 21 and 33 years old (*M* = 25.77, *SD* = 4.44). The study was approved by the local ethics committee of Georg-August-University Goettingen (reference number 240) and affirmed by the ethics committee of Friedrich-Alexander University Erlangen-Nürnberg (confirmation number 361_20 B) and conducted in line with the Declaration of Helsinki.

### Design

In a 2x2 within-participant design, the facial expression (positive and negative) and elicitation-method (posed and event-elicited) of the displayed stimulus were manipulated within participants. Mood was measured before and after the stimuli evaluation.

### Stimuli

The first onset frame (2^nd^ frame in each video clip) of the positive and negative expression stimuli were evaluated. In total, 192 images were presented to all the participants in a randomized order. Additionally, three onset frames of previously excluded video clips (e.g., because of later body movement) were shown and used as test trials. The stimuli were presented at the center of the screen with a default video width of 768 and height of 432 pixels.

### Materials and measurements

A 5-point scale was used to measure changes in mood before the evaluation of the stimuli and directly after the evaluation (1 = very sad, 5 = very happy). For valence ratings a neutral-midpoint scale was used (perceived valence: −7 = completely negative; 0 = don’t know; +7 = completely positive). Dawel et al. (2017) used such a neutral-midpoint scale to rate genuineness [21]. To keep the ratings comparable with the later validation study (study 2), the same rating scale was used for the valence ratings, only the anchors were adapted. Certainty in the valence rating was measured using a slider scale from 0% to 100% (0% = not sure at all, 100% = completely sure), accompanied by a visual analogue scale. For the intensity ratings a 10-point scale (0 = not at all intense; 1 = slightly intense; 9 = very intense) was used, as was the case in Dawel et al. (2017) [21]. The anchors were adapted to the German language and to be fitting to the rating question (Wie intensiv wirkte der Gesichtsausdruck des letzten Videos? 0 = Gar Nicht, 1 = Leicht, 9 = Sehr). The reported English anchors have been slightly relabelled compared to Dawel et al. (2017) [21], as those reported labels are more similar to the used German labels. A rating of zero was needed due to the inclusion of neutral stimuli.

### Procedure

**Fig 4:**
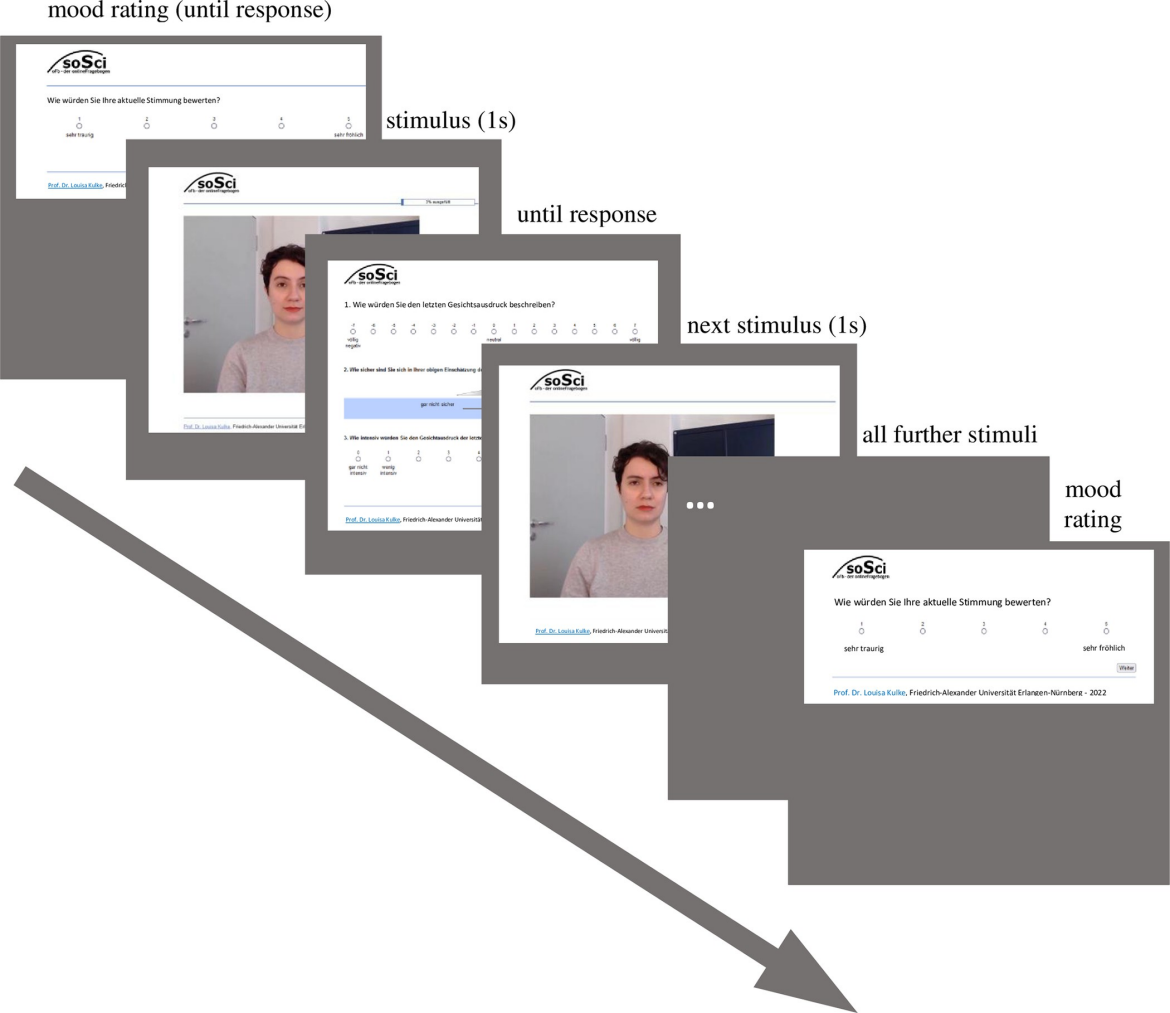
Experimental flow of study 1 and study 2.

The experiment was conducted as an online study on the platform SoSci Survey [55]. After digital informed consent was obtained, sociodemographic data was collected (age, gender, occupation, handedness, native language) followed by an explanation of the three rating questions and the procedure of the study. Lastly, a test trial followed consisting of three onset frames of prior excluded stimuli. After the test trials, the participants were informed of the start of the survey and the experiment started. Following the mood scale, the 192 positive and negative first onset frames were displayed in a randomized order with the three rating questions on the next page. Fig 4 depicts the experimental flow.

### Data preparation

Data was pre-processed in RStudio (version 4.2.0, [56]). We split the ratings for positive and negative expression stimuli in order to identify outliers in both expression valences. One rating was marked as extreme and therefore excluded. To investigate whether mood had an effect on the participants’ ratings, the means between pre and post mood were calculated for each participant and saved in an individual sheet.

## Materials and methods—Study 2: Dynamic stimuli validation

### Participants

All methods and analyses were in line with the preregistration (https://osf.io/cytrs) unless otherwise noted. Sample size calculations for emotional expression effects were conducted in RStudio (version 3.1.9.2, [56]) and a minimum sample size of 34 participants was determined and preregistered. Due to the length of the study, the stimuli were split into two stimulus sets. Therefore, we updated the sample size to 68, in order to have each stimulus rated by 34 participants. Participants were recruited via online advertisements and local databases. During the recruitment period, 78 participants completed the online study. Four of these participants had to be excluded since they did not fit into the age restrictions, and two more because they reported a diagnosed psychiatric or neurological disorder. Two participants had to be excluded due to technical errors. One more participant was excluded due to giving systematic answers to the rating questions. The last participant who completed the study was excluded from the analysis due to already having reached the preregistered sample size. After exclusions, sixty-eight participants (45 female, 23 male; 65 German speakers and 3 English speakers) between age 18 and 35 years (*M* = 25.26, *SD* = 3.66) were part of the data analysis. All participants had normal (35) or if short-sighted corrected-to-normal vision (33). The study was approved by the local ethics committee of Georg-August-University Goettingen (reference number 240) and affirmed by the ethics committee of Friedrich-Alexander University Erlangen-Nürnberg (confirmation number 361_20 B) and conducted in line with the Declaration of Helsinki. Digital written informed consent was obtained from each participant before the experiment. All personal information was saved separately from the other data. Each participant created a personal code so the experiment would remain anonymous. All 78 participants that made it to the end of the questionnaire were able to register for a raffle in which five of them received a voucher for Wunschgutschein.de valued at four times 15€ and one time 40€.

### Design

The study was planned as a 2x3 within-participants design with elicitation-method (posed and event-elicited condition) and facial expression (positive, neutral and negative) being manipulated within participants. Additionally, due to the length of the study, the stimuli were sorted into two sets (set 1, set 2), which were counterbalanced between participants. Participants’ mood was measured directly before and after the stimulus evaluation.

### Stimuli

In total, 294 dynamic facial expression stimuli had been created and standardized and were evaluated in the current study. The stimuli were standardized as described above. The stimuli were split in two sets and each participant was randomly chosen for one out of two stimulus sets, showing half of the dynamic stimuli. In addition, we used catch clips in the validation procedure to check whether the participants were able to spot edited video clips. Therefore, each participant evaluated 156 video clips (56 neutral, 41 positive, 50 negative, 5 positive catch clips, 4 negative catch clips). While the amount of positive, negative and neutral expression stimuli matched between the two stimulus sets, the amount per elicitation condition could not be matched exactly, nor could the amount of positive, negative and neutral stimuli be matched, due to necessary exclusions of video clips for standardization purposes. For the exact amounts see S2 Table. All clips were presented in a randomized order. The stimuli were presented in the middle of the screen at width = "768" and height = "432" pixels. The final dynamic stimuli can be found at the Open Science Framework platform (https://osf.io/xzfhr/).

### Materials and measurements

The same 5-point scale was used to measure changes in mood as in the earlier onset frame survey. For perceived valence, a similar scale was used as before. The valence slider scale consisted of 15 static points, but only the anchors were kept visible. For perceived intensity, the same rating scale was employed. Perceived genuineness and perceived edits of the stimuli were relevant in this validation study. For perceived genuineness a neutral-midpoint scale was used (perceived genuineness: −7 = completely fake; 0 = don’t know; +7 = completely genuine) based on Dawel et al. (2017) [21]. Only the anchors had to be adapted to German labels (Wie authentisch wirkte der Gesichtsausdruck des letzten Videos? -7 = völlig künstlich, 0 = weiss nicht, +7 = völlig authentisch). For perceived edits a yes-no question was asked to investigate whether the video stimuli seem like they have been edited or not.

### Procedure

The general procedure was similar to that of study 1, and we report all differences here. Before the study began, the participants could choose the language (English or German) of the experiment. Following given digital informed consent, the participants were randomly chosen for one stimulus set. Subsequently, sociodemographic questions were asked and the rating scales were explained. The German and English explanations of the rating scales are reported in the supplementary material (S2 File for German; S3 File for English). The explanations were followed by four test trials, consisting of three test video clips and one catch clip. After each quarter of displayed and rated video clips a break page was included. Participants could choose themselves when they wanted to continue. Fig 4 depicts the experimental flow of both online studies.

### Data preparation

The data of study 2 was prepared for analyses in the same manner as the data of study 1. Participants were excluded if they failed to complete the online study (*n* = 32), if technical errors occurred (*n* = 1), or if they did not fit the inclusion criteria (*n* = 6). Participants’ responses were checked for systematic ones (e.g., if most stimuli received the same valence rating), and participants were excluded if that was indicated (*n* = 1). Next, the mean rating of each stimulus was computed for every rating dimension (valence, intensity, perceived genuineness, and perceived edits). Afterwards, the participants’ mean ratings were checked for outliers. No extreme outlier ratings, outlier stimuli, or outlier participants were found, so no further data was excluded.

### Statistical analyses of studies 1 and 2

All statistical analyses were conducted in RStudio [56]. First, assumptions of the used statistical tests were checked as required in both studies, using central limit theorem, the Shapiro-Wilk normality test, histograms, and Q-Q-plots using the R package “ggplot2” [57]. All detailed assumption tests can be found in the S4 File. If not reported otherwise, the assumptions were met, and t-tests were conducted using the t.test function [56]. Otherwise, non-parametric equivalents were computed. For directional tests, two-tailed *p*-values are reported with a cut-off value of *p* < .10, and for non-directional tests *p* < .05 was used. Cohen’s d, the Mann-Whitney U test and its effect size was computed using the “rstatix” package [58], if that was not possible the effect size was calculated following Field and colleagues’ guide [59]. Bayes Factors (*BF*_*10*_) were calculated using the “BayesFactor” package [60].

For study 2 additional data processing was conducted using the package “matrixStats” [61]. First, due to the stimuli being split into two stimulus sets, the potential effects of stimulus set on the respective variables were determined either by computing a one-way analysis of variance (ANOVA) or a mixed ANOVAs using the aov function [56] for each hypothesis. Levene’s test of Equality of Variances was conducted using the “car” package [62]. If the ANOVA assumption were not met, a Kruskal-Wallis test was computed as a non-parametric alternative. The “rstatix” package was used to compute the respective effect size as well as the post-hoc Dunn’s test [58]. If no significant effect of stimulus set or an interaction with the respective variable of the hypothesis was found, t-tests were performed for the ratings of all stimuli. Otherwise, separate t-tests were performed.

If not otherwise specified, the used functions are part of RStudio’s “stats” package [56].

## Results—Development of the facial expression stimulus set

After the facial expression video recordings, the confederate answered four open questions regarding the emotion elicitation. The confederate stated that she could imaging the described situations well for most of the time, but could not imagine them for the duration of the whole video condition. She estimated that for a third of the time she could not imagine the situations. During the posed expressions she reported that she thought of nothing. The mean positive and negative affect scores during each elicitation condition can be seen in Table 2. Overall the mean removed frames for positive expression were 8.61 (*SD* = 6.15), whereas 6.40 (*SD* = 5.82) frames were removed for negative expressions (see Table 1). 0.55 (*SD* = 0.96) frames have been added overall for positive expression, while 2.41 (*SD* = 3.03) have been added for negative expressions. In Table 3 the mean edits per elicitation method can be seen.

**Table 2:** Mean positive and negative affect score of the confederate during the video recording.

Expression	Elicitation	Positive Affect Score	Negative Affect Score	
		*M (SD)*		*M (SD)*
Positive				
	Posed	16.67 (1.53)		11.33 (1.15)
	Event-Elicited	18.50 (0.71)		10.5 (0.71)
Negative				
	Posed	12.50 (2.12)		12.50 (0.71)
	Event-Elicited	16.50 (2.12)		14.00 (2.83)
Neutral				
	Posed	12.25 (0.96)		14.75 (3.59)
	Event-Elicited	17.00 (2.83)		11.5 (0.71)

**Table 3:** Mean removed and added frames for positive and negative expressions and the two elicitation methods.

Expression	Elicitation Method	Frames Removed Overall	Frames Added Overall	Frames Removed due to Blinking	Frames Removed to Create Clear Onset	Edits to Match Peak Times	
						Removed	Added
		*M (SD)*	*M (SD)*	*M (SD)*	*M (SD)*	*M (SD)*	*M (SD)*
Positive							
	Posed	8.38 (6.15)	0.38 (0.96)	3.40 (3.95)	0.58 (1.10)	4.40 (4.97)	0.38 (0.96)
	Event-Elicited	8.88 (4.98)	0.75 (1.53)	4.96 (3.63)	1.45 (3.73)	2.60 (3.05)	0.75 (1.53)
Negative							
	Posed	7.49 (6.66)	2.13 (3.15)	4.56 (4.66)	1.38 (3.55)	1.49 (2.41)	2.13 (3.15)
	Event-Elicited	5.13 (4.41)	2.74 (2.88)	3.96 (3.93)	0.60 (1.58)	1.57 (1.16)	2.74 (2.88)

*M* indicates mean. *SD* indicates standard deviation

## Results–Summary of study 1: First onset frame validation

In study 1, the participants rated the first onset frame of the developed positive and negative stimuli on three rating scales, namely valence, certainty and intensity. The detailed analyses can be found in the S5 File. Overall, the first onset frames of positive expression stimuli received a positive valence rating (*M* = 2.27, *SD* = 2.16), while the first onset frames of negative expression stimuli received a negative valence rating (*M* = -1.63, *SD* = 1.86). Further, the valence ratings of positive and negative expression stimuli differed significantly from each other, and both differed significantly from a neutral valence rating of zero. Moreover, for both positive and negative expressions, the event-elicited expressions received more extreme valence ratings than the posed expressions. For positive expression stimuli the mean *intensity* rating of the first onset frame was *M* = 4.63 (*SD* = 2.01), and for negative expression stimuli it was *M* = 3.93 (*SD* = 2.05). Further, the first onset frames of event-elicited stimuli were perceived as more intense than those of posed stimuli, for both positive and negative expression stimuli. *Mood* overall declined slightly during the survey with a mean rating of 3.39 (*SD* = 0.96) before the stimuli rating task and 3.31 (*SD* = 0.63) after.

## Results–Study 2: Dynamic stimuli validation

### Descriptive statistics

The mean ratings of each rating dimension for the three different expressions can be seen in Table 4. The mean ratings of study 1 and study 2 for each positive and negative expression stimulus included in the stimulus set can be seen in the S3 Table. The mean ratings of study 2 for neutral expression stimuli are reported in the supplementary material as well (S4 Table). Mood declined slightly during the study with a mean rating of 3.13 (*SD* = 1.67) before the stimuli rating task and 3.00 (*SD* = 0.77) afterwards, but the change was not significant, *t*(67) = 0.66, *p* = .51, 95% CI[-0.27, 0.53], *d* = .10, *BF*_*10*_ = 0.16. For positive expression catch clips the mean value for perceived edits was 1.86 (*SD* = 0.35), for negative expression catch clips it was 1.84 (*SD* = 0.37).

**Table 4:** Mean ratings and standard deviations of valence, intensity, genuineness and perceived edits for each expression.

Rating Dimension	Positive Expression Stimuli		Neutral Expression Stimuli	Negative Expression Stimuli
	*M (SD)*	*M (SD)*		*M (SD)*
Valence	0.95 (3.32)	0.13 (2.66)		-0.47 (3.06)
Intensity	4.10 (2.75)	3.02 (2.67)		3.71 (2.58)
Genuineness	1.65 (3.80)	2.23 (3.40)		2.00 (3.37)
Perceived Edits	1.87 (0.33)	1.88 (0.32)		1.88 (0.33)

*M* indicates mean. *SD* indicates standard deviation. Valence was rated on a 15-point scale (−7 = completely negative; 0 = neutral; +7 = completely positive). Intensity was rated on a 10-point scale (0 = not at all intense; 1 = slightly intense; 9 = very intense). Genuineness was rated on a 15-point scale (−7 = completely fake; 0 = don’t know; +7 = completely genuine). Perceived edits was a yes-no question, with yes indicating the perception of made edits (1 = No, 2 = Yes).

### Hypotheses testing

#### Valence perception of the different facial expressions

Valence ratings for negative expression stimuli were significantly lower than zero (i.e., neutral), which supported our hypothesis that negative expression stimuli are perceived as negative (see Table 5).

**Table 5:** One sample t-test statistics of valence ratings of positive and negative expression stimuli.

Expression	*Mean* (*SD*)	*t*-statistic	*df*	*p*	*d*	*BF*_*10*_
Negative	-0.47 (3.06)	-9.01	3399	< .001	-0.15	> 1.000.000
Positive	0.95 (3.32)	15.08	2787	< .001	0.29	> 1.000.000

The valence ratings were compared to a valence rating of zero, i.e., a neutral valence rating.

A one-way ANOVA revealed no significant main effect of stimulus set on valence, *F*(1) = 3.48, *p* = .06, *η*^2^ = 0.001, *BF*_*10*_ = 0.26. As preregistered, a one sample t-test was conducted for both stimulus sets together. The result revealed that positive expression stimuli received significantly higher *valence* ratings than a rating of zero (i.e., neutral), which supported our hypothesis that positive expression stimuli would be perceived as positive (see Table 5).

**Fig 5:**
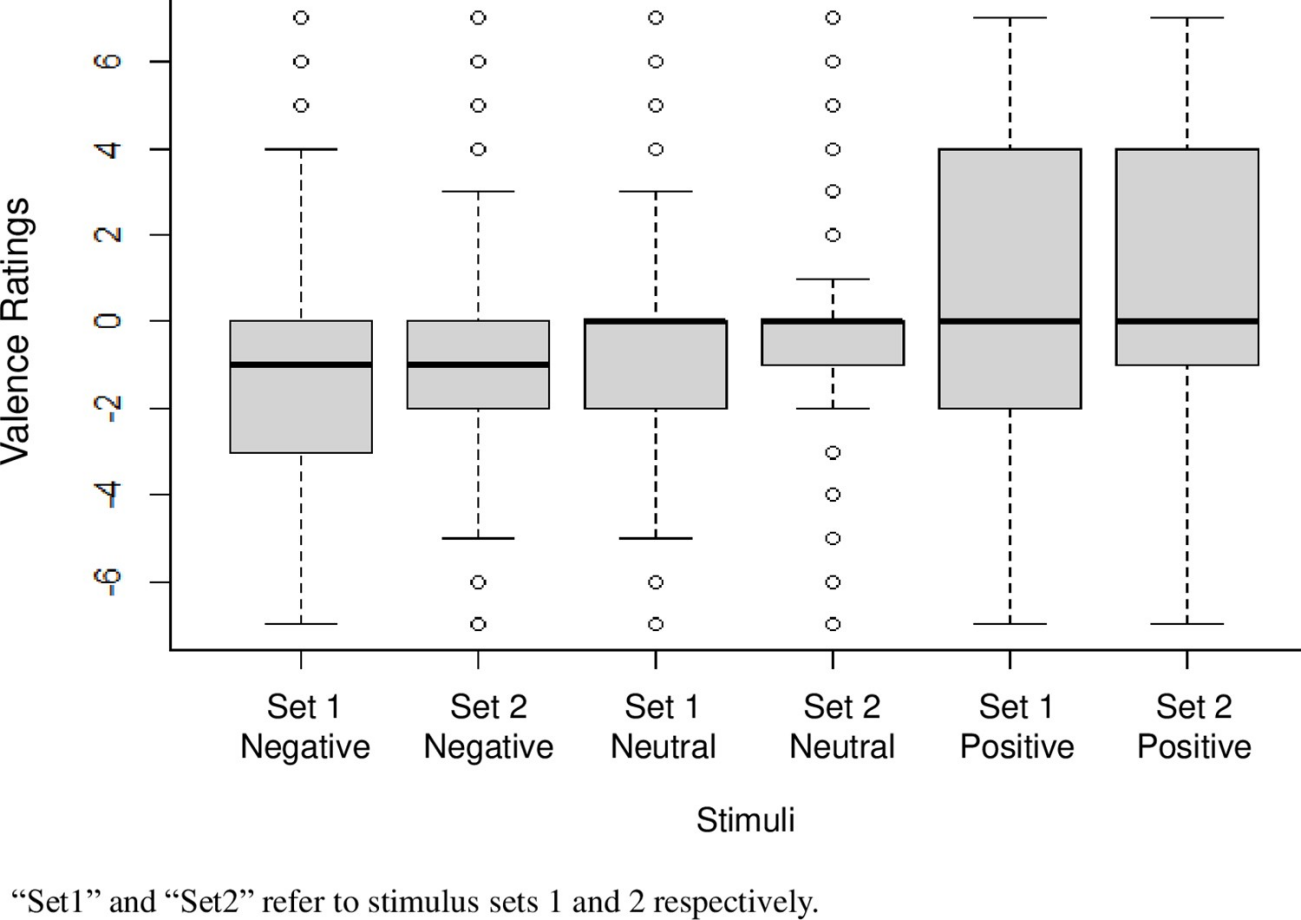
Valence ratings for negative, neutral and positive expression stimuli.

Hypothesis 3 stated that *valence* ratings of neutral videos should lie between negative and positive videos, significantly differing from both. Kruskal-Wallis test revealed a significant difference between the two stimuli tests (*p* < .001), therefore the following test were conducted separately for the two stimulus sets. First, the preregistered t-tests were computed, afterwards non-parametric equivalents were conducted as these were indicated due to the data not being normally distributed. Two paired t-tests were preregistered, however only independent t-tests could be calculated due to the different amounts of video clips per expression. In stimulus set 1 significant differences between neutral and positive, as well as neutral and negative expression stimuli were found (see Table 6). As was the case in stimulus set 2 for both neutral and positive, as well as neutral and negative expression stimuli (see Table 6). As mentioned above, non-parametric tests were computed as well. Mann-Whitney U tests were computed separately for each stimulus set to compare neutral with positive expressions, and neutral with negative expressions. We found significant differences in both sets between neutral and positive, as well as neutral and negative expression stimuli (see Table 7). Neutral expression stimuli received *valence* ratings that differed significantly from negative and positive expression stimuli in both stimulus sets. However, the medians were not conclusive for the comparison with positive stimuli, therefore one-sided tests were conducted to investigate whether positive expression stimuli were rated with a higher valence rating than neutral ones. For stimulus set 1, *U*(*n*_*neutral*_ = 1904, *n*_*positive*_ = 1394) = 1167619, *Z* = -6.05, *p* < .001, *r* = 0.11, and for stimulus set 2, *U*(*n*_*neutral*_ = 1904, *n*_*positive*_ = 1394) = 1162730, *Z* = -6.22, *p* < .001, *r* = 0.11, a significant result was found, which supported our hypothesis. In Fig 5, the valence ratings of negative, neutral and positive expression stimuli can be seen for both stimulus set 1 and 2. *To conclude*, *the valence ratings of neutral expression stimuli did lie between those of negative and positive expression stimuli in both stimulus sets*. *This was revealed by both the preregistered parametric and the additional non-parametric statistical analyses*.

**Table 6:** Independent t-test statistics of valence ratings of neutral and positive and neutral and negative expression stimuli for each stimulus set.

Stimulus Set	Expression	M (SD)	*t-statistic*	*Df*	*p*	*d*	*BF*_*10*_
Set 1							
	Neutral	0.03 (2.71)					
	Positive	0.83 (3.35)	-365.62	*7478972*	< .001	-0.26	> 1.000.000
	Negative	-0.58 (3.10)	292.44	*7670581*	< .001	0.21	> 1.000.000
Set 2							
	Neutral	0.22 (2.60)					
	Positive	1.07 (3.28)	-397.88	*7418224*	< .001	0.28	> 1.000.000
	Negative	-0.37 (3.01)	293.58	*7642996*	< .001	0.21	> 1.000.000

**Table 7:** Mann-Whitney U test statistics for comparing valence ratings of neutral and positive and neutral and negative stimuli.

Stimulus Set	Expression	*Median*	*n*	*U-statistic*	*Z*	*p*	*r*
Set 1							
	Neutral	0.00	1904				
	Positive	0.00	1394	1167619	6.05	< .001	0.11
	Negative	-1.00	1700	1343416	-9.03	< .001	0.15
Set 2							
	Neutral	0.00	1904				
	Positive	0.00	1394	1162730	-6.22	< .001	0.11
	Negative	-1.00	1700	1329036	-9.49	< .001	0.16

#### Genuineness and intensity perception of the different facial expressions

The standard deviations of *genuineness* ratings were explored by computing the standard deviation for all video clips of one expression category, separately for each participant. A mixed ANOVA was conducted with stimulus set as the between-subject factor and expression as the within subject factor. A statistically-significant difference in *genuineness* was not found for stimulus set, *F*(1) = 0.70, *p* = .40, *η*^2^ = .001, *BF*_*10*_ = 0.21, but for expression, *F*(2) = 8.19, *p* < .001, *η*^2^ = .04, *BF*_*10*_ = 66.55; however not for an interaction of stimulus set and expression, *F*(1,2) = 0.01, *p* = .99, *η*2 < .001, *BF*_*10*_ = 1.33. As the data of the two stimulus sets did not significantly differ from another, only two independent t-tests were conducted. No significant difference in standard deviations of *genuineness* ratings was found between negative (*M*_*SD*_ = 2.91, *SD*_*SD*_ = 0.87) and neutral (*M*_*SD*_ = 2.85, *SD*_*SD*_ = 0.85) expression stimuli, *t*(*133*.*89*) = 0.39, *p* = .70, 90% CI[-0.19, 0.30], *d* = 0.07, *BF*_*10*_ = 0.20. A significant difference in standard deviations of *genuineness* ratings was however revealed between negative and positive (*M*_*SD*_ = 3.41, *SD*_*SD*_ = 0.94) expression stimuli, *t*(*133*.*27*) = -3.25, *p* = .001, 90% CI[-0.76, -0.25], *d* = -0.56, *BF*_*10*_ = 20.41. However, against expectations, the mean values of the standard deviations indicated that the *genuineness* ratings of positive expression stimuli varied more than those of negative expression stimuli, which contradicts our hypothesis. *To conclude*, *the genuineness ratings of negative and neutral expression stimuli varied similarly*, *while the genuineness ratings of positive expression stimuli varied significantly more than those of negative ones*.

**Fig 6:**
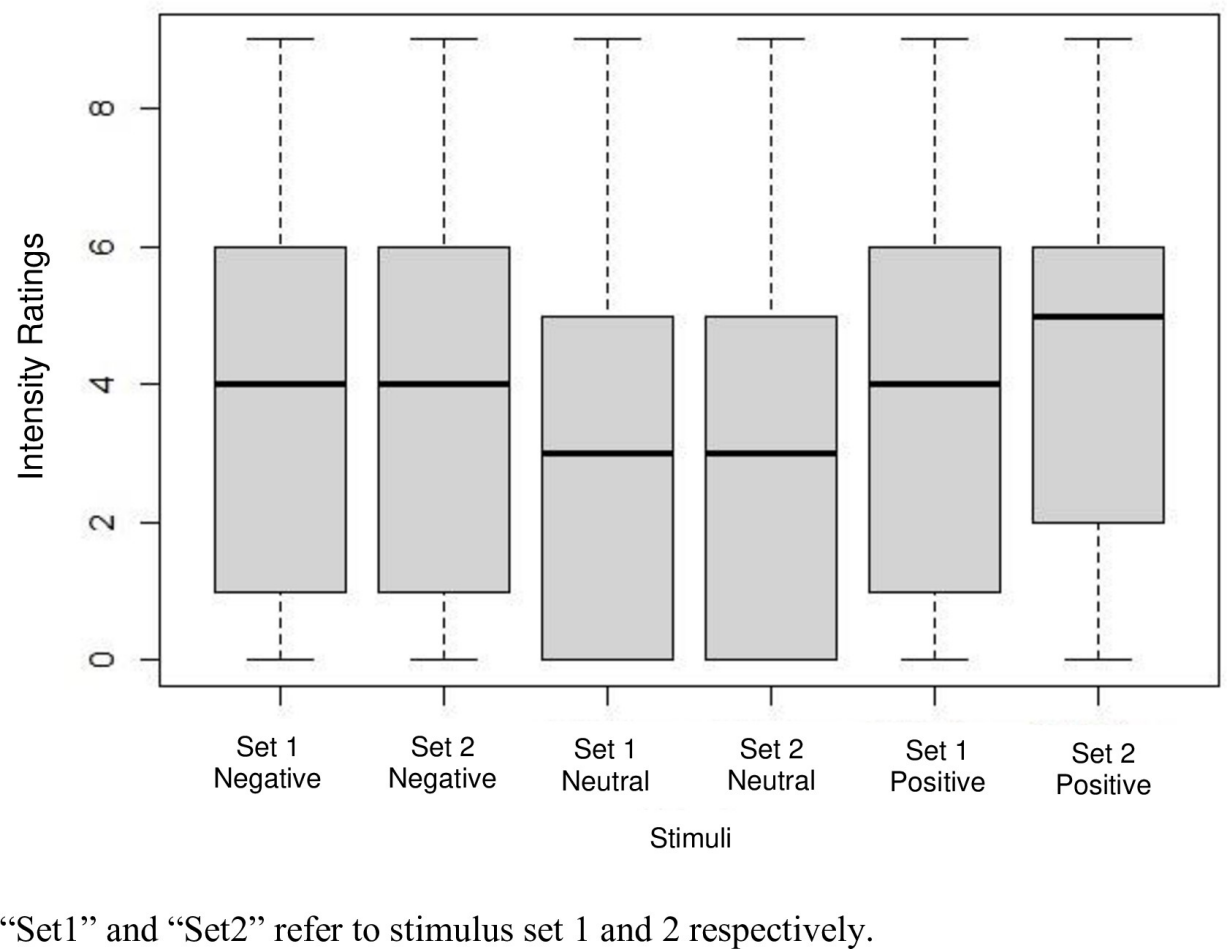
Intensity ratings of negative, neutral and positive expression stimuli in stimulus set 1 and stimulus set 2.

In Fig 6, the intensity ratings of negative, neutral and positive expression stimuli are displayed. The mean *intensity* rating of positive expression stimuli was 4.10 (*SD* = 2.75), and for negative expression stimuli it was 3.71 (*SD* = 2.58). Kruskal-Wallis tests revealed significant results for both, the stimulus set, *H*(1) = 5.24, *p* = .02, *η*^2^ < 0.001, and expression, *H*(2) = 33.61, *p* < .001, *η*^2^ = 0.005. Due to a significant difference between the two stimulus sets, the following t-tests were conducted separately for each stimulus set. Once again, the preregistered paired t-test could not be computed due to the different numbers of video clips of each expression. The independent t-tests resulted for both stimulus set 1, *t*(2884.1) = -3.35, *p* < .001, 90% CI[-0.49, -0.17], *d* = -0.12, *BF*_*10*_ = 11.78, and stimulus set 2, *t*(2906.6) = -4.80, *p <* .*001*, 90% CI[-0.62, -0.30], *d* = -0.17, *BF*_*10*_ = 4142.80, in highly significant differences in *intensity* ratings of positive and negative expression stimuli. The means, however, indicated that the *intensity* of positive expression stimuli was rated higher than that of negative expression stimuli. Exploratorily, this was tested with one-tailed independent t-tests, showing significant results for both stimulus set 1, *t*(2884.1) = -3.35, *p* < .001, *d* = -0.12, *BF*_*10*_ = 11.78, and stimulus set 2, *t*(2906.6) = -4.80, *p <* .*001*, *d* = -0.17, *BF*_*10*_ = 4142.80. *Therefore*, *the results did not support the fifth hypothesis*, *since the intensity of positive expression stimuli was rated higher than the intensity of negative ones*.

Hypothesis 6 stated that the video clips rated as low to moderate intensity (0–4) are considered more genuine than the video clips rated as moderate to high intensity (5–9). To investigate this, the mean *genuineness* ratings were computed for those video clips rated low to moderate in intensity (*M*_*Intensity*_ = 3.30, *SD*_*Intensity*_ = 0.47) and those rated moderate to high in intensity (*M*_*Intensity*_ = 4.33, *SD*_*Intensity*_ = 0.25). For low-intensity stimuli, the mean *genuineness* rating was 2.08 (*SD* = 0.57), while the mean *genuineness* rating for high-intensity stimuli was 1.70 (*SD* = 0.76). Kruskal-Wallis tests were computed and revealed significant results for stimulus set, *H*(1) = 11.11, *p* < .001, *η*^2^ = 0.03, expression, *H*(1) = 29.16, *p* < .001, *η*^2^ = 0.09, and intensity level, *H*(1) = 12.31, *p* < .001, *η*^2^ = 0.04. A pairwise post-hoc Dunn’s test with Bonferroni adjustments revealed significant differences in intensity levels of all three expressions, *p* < .001. Due to a significant difference between the two stimulus sets, the follow-up tests were again conducted separately. The preregistered t-tests revealed significant results for both stimulus set 1, *t*(45.86) = 3.07, *p* = .004, 90% CI[0.18, 0.60], *d* = 0.68, *BF*_*10*_ = 43.95, and stimulus set 2, *t*(52.55) = 2.48, *p* = .02, 90% CI[0.12, 0.59], *d* = 0.54, *BF*_*10*_ = 8.25, showing that *genuineness* ratings were greater for low intensity stimuli than for high intensity stimuli. To follow up possible explanations, neutral expression stimuli were excluded. Mean values also indicated that neutral expression stimuli received the lowest *intensity* ratings (*M* = 3.02, *SD* = 0.44), and the highest *genuineness* ratings (*M* = 2.23, *SD* = 0.57). Whereas before the sample size of low intensity stimuli (*n*_*set1*_ = 113, *n*_*set2*_ = 108) was more than double the size of the sample size of high intensity stimuli for both stimulus sets (*n*_*set1*_ = 34, *n*_*set2*_ = 39), the sample sizes became more equal after the exclusion of the neutral expression stimuli. Kruskal-Wallis test indicated a significant difference between the stimulus sets once more, *H*(1) = 5.76, *p* = .02, *η*^2^ = 0.03. Consequently, the following independent t-tests were conducted separately for each stimulus set. For stimulus set 1, a significant difference in *genuineness* for low and high intensity stimuli was revealed again with a moderate effect size, *t*(53.28) = 2.13, *p* = .04, 90% CI[0.06, 0.51], *d* = 0.50, *BF*_*10*_ = 2.22. However, for stimulus set 2 the difference between low and high intensity stimuli did no longer reach significance, *t*(61.94) = 1.31, *p* = 0.19, 90% CI[-0.05, 0.45], *d* = 0.29, *BF*_*10*_ = 0.52. *The results above support the hypothesis that low intensity stimuli are perceived as more genuine than high intensity ones*. *However*, *when only comparing low and high intensity positive and negative expressions*, *the results were less distinct*. *No significant difference between low and high intensity expressions was reached on stimulus set 2*, *but a significant moderate effect size could still be found in stimulus set 1*.

#### Influence of the elicitation method on genuineness perception

Hypothesis 7 stated that there is not a significant difference in *genuineness* ratings between posed video clips (*M* = 1.92, *SD* = 3.61) and event-elicited video clips (*M* = 1.31, *SD* = 3.99) that are displaying positive expressions. Kruskal-Wallis test showed a significant difference in *genuineness* ratings between the two stimulus sets, *H*(1) = 5.99, *p* = .01, *η*^2^ = 0.002. Therefore, the hypothesis was tested separately for both stimulus sets. Instead of the preregistered paired t-tests, independent t-tests had to be computed due to the differing numbers of observations. For stimulus set 1, the mean *genuineness* rating for posed stimuli 1.95 (*SD* = 3.61) and for event-elicited stimuli 1.63 (*SD* = 4.01) did not significantly differ, *t*(1308.8) = 1.54, *p* = 0.12, d = 0.08, *BF*_*10*_ = 0.20. For stimulus set 2, the mean *genuineness* rating for posed stimuli 1.89 (*SD* = 3.62) and for event-elicited stimuli0.97 (*SD* = 3.93) significantly differed, *t*(1257.7) = 4.47, *p* < .001, *d* = 0.24, *BF*_*10*_ = 1334.87. We explored whether all stimuli were perceived as genuine, i.e., if the *genuineness* ratings were significantly above zero. As there was not a significant difference between posed and event-elicited expressions of positive expression stimuli for stimulus set 1, a one-sample t-test was computed to compare the *genuineness* ratings with zero. The result revealed that the *genuineness* ratings were significantly above zero, which means the positive expression stimuli in stimulus set 1 were perceived as genuine, *t*(1393) = 17.7, *p* < .001, *d* = 0.47, *BF*_*10*_ = 6003683x10^53^. As there was a significant difference between posed and event-elicited positive expression stimuli in stimulus set 2, two one-sample t-tests were conducted. For both the posed, *t*(781) = 14.6, *p* < .001, *d* = 0.52, *BF*_*10*_ = 2066242 x10^33^, and the event-elicited positive expression stimuli of stimulus set 2, *t*(611) = 6.12, *p* < .001, *d* = 0.25, *BF*_*10*_ = 3259800. Therefore, the positive expression stimuli were on average perceived as genuine. *The sixth hypothesis was partially supported by the results*, *while there was no significant difference between posed and event-elicited positive expressions in stimulus set 1*, *significance was reached in stimulus set 2*. *Furthermore*, *the posed stimuli were perceived as significantly more genuine as the event-elicited ones in stimulus set 2*, *as the mean values indicated*. *However*, *the exploratory analyses revealed that nonetheless posed and event-elicited positive expression stimuli were overall perceived as genuine*.

Hypothesis 8 stated that the event-elicited video clips (*M* = 2.02, *SD* = 3.32) displaying a negative expression are rated as more genuine than the posed video clips (*M* = 1.99, *SD* = 3.41) displaying a negative expression. A mixed ANOVA with stimulus set as between-subject factor and elicitation method as within subject-factor, revealed no main effect of stimulus set, *F*(1) = 3.72, *p* = 0.05, *η*^2^ < 0.001, *BF*_*10*_ = 0.25, nor of elicitation method, *F*(1) = 0.03, *p* = 0.87, *η*^2^ < 0.001, *BF*_*10*_ = 0.04, and no interaction between the two either, *F*(1) = 0.02, *p* = 0.90, *η*^2^ < 0.001, *BF*_*10*_ = 0.01. Consequently, one t-test was computed to compare *genuineness* ratings of posed and event-elicited negative expression stimuli. Due to the different numbers of observations an independent one instead of the preregistered paired t-test had to be computed. However, no significant result was revealed, *t*(3298) = 0.21, *p* = .84, 90% CI[-0.17, 0.21], *d* < 0.001, *BF*_*10*_ = 0.04. We investigated exploratorily whether all stimuli were perceived as genuine, that is if the genuineness ratings were significantly above zero. The one-sided one-sample t-test revealed that the negative expression stimuli (*M* = 2.00, *SD* = 3.37) differed significantly from zero, *t*(3399) = 34.6, *p* < .001, *d* = 0.59, *BF*_*10*_ = 2279101x10^215^, indicating that they were perceived as genuine. *Hypothesis 8 could not be supported by the findings of the current study as posed and event-elicited negative expression stimuli did not differ in perceived genuineness*, *but negative expression stimuli were perceived as genuine*.

Hypothesis 9 stated that there was no difference in *genuineness* ratings between posed (*M* = 2.29, *SD* = 3.39) and event-elicited (*M* = 2.16, *SD* = 3.41) neutral expression video clips. Kruskal-Wallis test indicated significant differences between the stimulus sets, *H*(1) = 5.52, *p* = .02, *η*^2^ = 0.001. Thus, the two stimulus sets had to be tested separately. As the sample sizes of posed and event-elicited neutral stimuli were the same for stimulus set 1, the preregistered paired t-test was computed first, showing no significant difference in genuineness of posed (*M*_*set1*_ = 2.35, *SD*_*set1*_ = 3.31, *Mdn*_*set1*_ = 3.00) and event-elicited (*M*_*set1*_ = 2.37, *SD*_*set1*_ = 3.28 *Mdn*_*set1*_ = 3.00) neutral expression stimuli, *t*(951) = 0.22, *p* = 0.82, *d* = 16.54, *BF*_*10*_ = 0.05, as did the non-parametric Wilcoxon signed rank tests for stimulus set 1, *p* = .85, *r* = 0.004. The sample sizes in stimulus set 2 were not equal, because one more posed expression and one less event-elicited expression were included in the stimulus set. First, instead of the preregistered paired t-test, an independent one was computed to compare the genuineness of posed (*M*_*set2*_ = 2.45, *SD*_*set2*_ = 3.47, *Mdn*_*set2*_ = 3.00) and event-elicited (*M*_*set2*_ = 1.93, *SD*_*set2*_ = 3.53, *Mdn*_*set2*_ = 2.00) neutral expression stimuli, which revealed a significant result, *t*(1886.7) = -1.96, *p* = 0.0497, *d* = -0.09, *BF*_*10*_ = 0.35, as did the indicated Mann-Whitney U test, *U(n*_*posed*_ = 986, *n*_*event-elicited*_ = 918) = 428889, *Z* = -1.98, *p* = .047, *r* = 0.05. Three one-sided Wilcoxon signed rank test were computed to compare the *genuineness* ratings of neutral expression stimuli with zero. One was conducted for the neutral expression stimuli of stimulus set 1, *p* < .001, *r* = -0.57. Two were computed for the neutral expression stimuli of stimulus set 2, for the posed, *p* < .001, *r* = -0.55, and the event-elicited, *p* < .001, *r* = -0.48, neutral expression stimuli of stimulus set 2. Therefore, the neutral expression stimuli were overall rated as genuine, as well. *The findings above partially support hypothesis 9 as a significant difference between posed and event-elicited neutral expression stimuli could be found in stimulus set 2*, *however only with a small effect size*. *Nonetheless*, *the neutral expression stimuli were perceived as genuine on average*.

## Discussion

The current set of studies aimed to develop and validate a set of standardized natural dynamic facial expression stimuli. Within the validation of these stimuli, differences in the perception of valence and intensity of the first onset frames were investigated, as well as the perception of valence, intensity and genuineness of the dynamic facial expression stimuli. Moreover, the effects of two different elicitation methods of emotional facial expressions were explored.

### Development of the facial expression stimulus set

The natural dynamic facial expression stimuli were standardized in lighting, background, sitting position, clothing, face-to-video ratio, video length, as well as their dynamic aspects (onset and peak expression duration), blinking amount, and luminance. Regarding the dynamic aspects of the stimuli, positive and negative expression stimuli had to be edited more extensively. To achieve standardized positive and negative expression stimuli, certain frames had to be removed or added. For positive expression stimuli, significantly more frames were removed than for negative expression stimuli, however significantly more frames were added for negative expression stimuli than for positive ones. When comparing these two effect sizes (more removed frames for positive, more added frames for negative expression stimuli), it was evident that they were only slightly different. Therefore, the number of edits between the two kinds of emotional expression stimuli can be described as similar. As the amounts of removed and added frames give an indirect approximation for the onset durations, we can conclude that the onset duration was originally longer for positive expressions than for negative expressions. This is in line with past research. For instance Pollick et al. (2003) suggested an increased expression duration of 1100ms for happiness compared to 933ms for anger [63]. While Pollick et al. (2003) focused on overall duration [63], it is plausible that onset-duration would differ as well.

Moreover, previous research on the relationship between onset duration and genuineness perception found that happy expressions with longer onset-durations (i.e., 528ms) are perceived as more genuine that ones with a shorter onset-duration (132ms, or 231ms) [14]. Thus, standardizing the onset duration to 600ms should result in at least positive expressions that are perceived as genuine. Furthermore, Recio et al. (2013) suggested that positive and negative expressions with onset-durations from 200 to 500ms can be accurately recognized, whereas when expressions are shown faster or slower they tend to be confused with others [64]. One exception would be sadness, as this is an expression that enfolds more slowly [64]. While the results of Recio et al. (2013) do not directly indicate that expressions with these onset-durations will also be perceived as genuine [64], it is reasonable that the onset duration at which expressions are recognized best, is also the duration that seems most natural and therefore genuine. The results of study 2 are in line with these past findings.

### Study 1: First onset frame validation

During study 1 the first onset frames of the positive and negative expression stimuli were evaluated in terms of valence and intensity. For both, positive and negative expression stimuli the first onset frames were accurately recognized as positive and negative in valence and differed significantly from each other and from a neutral valence rating. Even though, positive, and negative expressions like e.g., happy and angry expressions have high recognition rates (e.g., Goeleven et al., 2008; Langner et al., 2010 [5, 31]), it was unclear if the same was the case when only the first onset frame of an expression is seen. Additionally, this finding also supports the success of the stimuli standardization, as both positive and negative expressions can be recognized from the first onset frame.

The intensity of the first onset frame was perceived as higher for positive expression stimuli compared to negative expression stimuli, however the effect size was only small. It is generally possible that the standardization has created more intense positive expression stimuli compared to negative ones, as more frames had to be removed for positive expressions, and therefore creating a faster evolving expression compared to the original positive expressions. However, frames have only been removed before the first onset frame if the expression in the first onset frame was unclear. Therefore, the standardization might not actually be the reason for the differing intensities of positive and negative expressions.

### Study 2: Dynamic stimuli validation

All dynamic stimuli were evaluated in regard to valence, intensity and genuineness in study 2. The dynamic stimuli displaying positive, neutral and negative expressions were accurately recognized in their intended valence, and differed significantly from each other in valence. However, not all positive expression stimuli were rated as positive and not all negative expression stimuli were rated as negative. These stimuli can be discarded when designing a study in which the stimuli are used. When comparing valence and intensity ratings of our stimuli to those ratings reported in previous literature on facial expression databases as done by e.g., Krumhuber and colleagues (2021), it is difficult to draw a clear conclusion as ratings of valence and intensity as well as naturalness varied depending on the rated stimuli database [65]. However, for those stimuli databases evaluated, deliberately posed expressions were perceived as more intense and higher intensity ratings positively predicted the participants’ level of recognition [65]. Opposite to that, the posed expressions in our stimulus set did not receive higher intensity ratings. In past research a valence scale ranging from 1 (very negative) to 9 (very positive) was often employed (e.g., Dawel et al., 2017 [21]). In the validation studies of Dawel and colleagues (2017) the NimStim set of facial expressions [30] and the Radbound Faces Database [5] were evaluated, the positive expressions of these databases received a mean rating of 7.78 and 7.43 respectively. As we used a 15-point valence scale with a neutral midpoint ranging from -7 (completely negative) to +7 (completely positive), it is difficult to compare these ratings. It is however evident that our positive stimuli (*M* = 0.95, *SD* = 3.32) received a mean rating closer to a neutral rating than the other databases did. This could be due to the main purpose of the present stimuli set to be natural and therefore possibly lower in both valence and intensity. Regarding intensity perception, neutral expression stimuli were perceived as lowest in intensity with a mean rating of low to moderate intensity, followed by negative expression stimuli with a mean rating of moderate intensity and finally positive ones which were perceived as highest in intensity with on average a moderate to high intensity rating. Neutral expression stimuli received the lowest intensity ratings, which is in line with previous research on the intensity of different facial expressions [22, 66]. The higher intensity of positive expressions stimuli is contrary to our expectations and the results of Biele and Grabowska (2006) [1]. However, they are in line with previous findings of Garrido et al. (2016) who reported higher intensity perception of smiling expressions compared to frowning ones [22]. While we did not label the displayed facial expressions, a frowning expression seems to be more appropriate as negative visual feedback than an angry expression. Consequently, it is reasonable that our negative expression stimuli were perceived as less intense than the positive expression stimuli, even though angry expressions are perceived as more intense than happy expressions [1]. Another explanation is given by the results of Hess et al. (1997), which indicated that high intensity of negative facial expressions is only perceived in negative facial expressions of male actors, and in positive facial expressions of female actors [66]. Since our model, the confederate, was female, this could have influenced the intensity perception of the different facial expressions.

Overall, positive, neutral and negative expression stimuli were all perceived as genuine. Furthermore, overall low-intensity expressions were rated as more genuine than high-intensity expressions, however when excluding all neutral expression stimuli from the calculation, the results were less distinct. While the effect size remained moderate for stimulus set 1 as well as significant, no significant difference was found in stimulus set 2. Nevertheless, the results still indicate a tendency for lower intensity expressions to be perceived as more genuine than higher intensity ones. As mentioned before, lower intensity expressions could be perceived as more genuine due to subtler expression being seen more often in everyday life [37], or because the confederate was better at displaying subtler expressions in a more genuine way.

In previous research, facial expression stimuli databases included different stimuli models and therefore describe individual’s characteristics such as perceived dominance (e.g., Sutton et al., 2019 [67]). Since our stimuli depict only one person, we chose not to include a dominance rating question. Our female model might possibly receive a lower dominance rating than a male model would, as previous research indicates rating differences based on the model’s gender (e.g., Carney et al., 2007 and Hess et al., 2000 [68, 69]); however, not only gender but also facial features as well as contextual factors contribute to the perception of dominance in facial expressions [70].

### Overall findings on the elicitation method of emotional facial expressions: Event-elicited vs. posed

Within the current studies no significant difference between posed and event-elicited expressions was found during the development of the standardized stimuli, however, significant differences between posed and event-elicited expressions were found in the two validation studies for valence, intensity, and genuineness.

Regarding the stimulus development, posed and event-elicited expressions did not differ regarding removed or added frames, neither for positive expression stimuli, nor for negative expression stimuli. In past research genuine and deliberate expressions differed in their overall durations, with e.g., deliberate smiles being shorter in their onset- and offset-times [14, 44, 71, 72]. Our result show no difference between the posed and event-elicited expressions, which diverges from past findings. However, the confederate was aware that the peak expression should be reached within one second (as the stimuli should be EEG compatible), which could have influenced the speed of the displayed expressions more than the elicitation methods did. Secondly, both the event-elicited and posed expressions were shown in a relatively deliberate and laboratory context, therefore none of the expressions did actually occur spontaneously. Thus, the equal durations of the expressions could indicate that the two elicitation conditions do not actually differ in their degree of genuineness.

Regarding the first onset frames (study 1), the event-elicited expression stimuli received more extreme valence and intensity ratings than the posed ones, i.e., event-elicited positive expression stimuli were rated as more positive and more intense, while event-elicited negative expression stimuli were rated as more negative and more intense than the posed expressions. As the amount of removed and edited frames did not differ between the elicitation conditions, the reason for this difference must lie somewhere else other than the standardization, possibly in the elicitation method. That would mean that the application of the two elicitation methods was successful in creating expressions that look differently. There are, however, alternative explanations as well, e.g., the procedure of the video recording could have caused a difference between the two differently elicited expressions, as all posed expressions were recorded before the event-elicited ones. Thus, for example the practice during the posed condition could have influenced the expressions in the event-elicited condition. However, we had decided that the event-elicited expressions should be recorded after all posed expressions, to not influence the posed expressions with the elicited emotions of the event-elicited condition.

Regarding the perceived genuineness of the dynamic stimuli, a tendency for posed positive and posed neutral expressions to be perceived as more genuine than their event-elicited counterparts emerged, which is inconsistent with prior research (e.g., Dawel et al., 2017 [21]). For negative expression stimuli, no significant differences in perceived genuineness between the elicitation methods were found. This finding does not support the notion that event-elicited expressions are automatically perceived as more genuine, which was assumed in the past (e.g., Garrido et al., 2016 [22]), neither does this finding support our hypotheses. Compared to stimuli used in past research on the perception of posed and spontaneous/event-elicited expressions, the standardization that our stimuli underwent differs (e.g., creating a clear first onset frame and matching the peak expression time). However, during the standardization process event-elicited positive expressions were edited in a similar way as the posed positive expression stimuli, and no significant differences in edits were revealed in the analyses. Therefore, the difference in perceived genuineness cannot be accounted to the standardization. An alternative explanation is the fact that we only used one model, and that we could not counterbalance the sequence that posed and event-elicited expressions were filmed. As mentioned above, we had decided that that the event-elicited expressions should be recorded after all posed expressions. So, when recording the event-elicited expressions, the confederate had already displayed each expression approximately 60 times, which might have influenced the facial expressions due to facial muscle tension or fatigue [73]. Because of the fatigue of the confederate’s facial muscles, it may have been more strenuous for the confederate to portray the same facial expression with similar intensity, which might have been visible and have caused the event-elicited expression to be perceived as more extreme in valence and intensity and as less genuine. Furthermore, the confederate was instructed to imagine that she is giving a participant positive, negative or no visual feedback to a task, however, in the current validation study, the video recordings were used regardless of whether the emotion elicitation was successful (as measured using the PANAS). This allows other researchers to freely choose stimuli for potential re-use.

### Findings on the use of the developed stimuli in neuroscientific research

The aim of the current set of studies was to develop and validate a standardized natural dynamic facial expression stimulus set. The stimuli were standardized in background, lighting, sitting position and face-to-video ratio, as well as luminance, and certain temporal aspects. For positive and negative expression stimuli, the duration of a neutral expression at the beginning was standardized to approx. 33ms (i.e., one frame), the duration of the onset of the respective expression to 600ms (i.e., 18 frames) and the duration of the peak expression to approx. 367ms (i.e., 11 frames), and the overall length of the video stimuli to one second. Furthermore, blinking was kept to a minimum, and eyes were open at the beginning and the end of each stimulus. The evaluations of the first onset frames showed that the intended valence of the expressions can already be recognized at that point. The intended valence was also accurately recognized when the dynamic stimuli were displayed. However, the intensity of positive expression stimuli was rated higher than the intensity of negative expression stimuli, for both the first onset frame as well as for the full dynamic stimulus. Overall, the stimuli were perceived as genuine. To conclude, the developed dynamic facial expression stimuli are standardized though as natural as possible. However, it is possible to control facial expression stimuli even more by using computer-morphing software, as was done in the past (e.g., Krumhuber & Kappas, 2005 and Recio et al., 2013 [14, 64]).

### Considerations when controlling natural stimuli

While the aim was to create stimuli that resemble natural facial expressions, different aspects limit the actual naturalness. The facial expressions were recorded in a laboratory context, even if the aim was to create posed as well as event-elicited emotional expressions, they were both shown in a deliberate context. Furthermore, the temporal aspects of the dynamic stimuli had to be controlled, and thus the durations of onset and peak have been altered. Additionally, certain movements like blinking were controlled by removing them from some stimuli. While all changes were kept minimal, they somewhat changed the naturalness of the stimuli. However, it should be noted that there is always a trade-off between experimental control and ecological validity of stimuli [5]. In this case we chose ecological validity.

Regarding the validation of the stimuli, in study 1 only the first onset frames of the positive and negative expression stimuli were evaluated, but it is unclear whether the neutral expression frame of the positive and negative expression stimuli is actually perceived as neutral. Due to manually determining the emotional onset of the expressions, it is possible that the chosen neutral expression frame, which is followed by the first emotional onset frame, is not completely neutral. Further, due to the block design of the video recording, the respective expressions were displayed multiple times in a row, which could have led to a difference the neutral face between the displays of the expressions. However, because of the study length, we could not include the neutral onset frames as well. Further, the use of only one model might have caused a habituation effect [74] and additionally, the use of only one model did not allow for an exploration of gender-specific effects in expression production [75].

In previous research, not only valence ratings were used, but also arousal ratings of emotional expression stimuli. According to Lang and colleagues (1998) and to Reisenzein (1994) valence refers to the qualitative component of emotions, ranging from negative to positive, while arousal refers to the intensity of emotions [23, 24]. These definitions were employed in previous research by Sato and Yoshikawa (2007) on emotional expression stimuli, as well [76]. In the current study we measured “intensity”, as a loose German translation of the word “arousal”, as this label would be easier to understand for untrained German raters.

### Implications

Despite the stated limitations, a set of standardized and yet natural as well as dynamic facial expression stimuli has been developed and validated. Most importantly, the facial expressions of the stimuli can be accurately recognized, are similar in intensity and perceived as genuine. Due to them also being standardized in length, duration of their dynamic aspects, movement, lighting, and blinking as well as luminance the stimuli are suitable for the use in neuroscientific research. Most research with dynamic facial expression stimuli has relied on computer-morphed stimuli, (e.g., Biele & Grabowska, 2006, Holland et al., 2019, Kamachi et al., 2013, Krumhuber & Kappas, 2005, Montagne et al., 2007, Recio et al., 2011, 2014 and Sato & Yoshikawa, 2004 [1, 12–18]), which is a more controlled approach to conduct research on emotional facial expression. As there is always a trade-off between experimental control and ecological validity of stimuli [5], the use of the current stimuli set would shift that trade-off more towards ecological validity.

## Conclusions

The application of facial expression stimuli in research, e.g., neuroscientific research, requires standardized and validated stimuli. Technical features such as background, lightning conditions, luminance, clothing, and focal distance play a role next to temporal aspects of dynamic stimuli, like onset and peak duration. The developed dynamic facial expression stimulus set provides adequate stimuli for this research field as they have been standardized both in technical and in temporal characteristics. Researches can choose the stimuli that received valence, intensity and/or genuineness ratings that fit their research aims best. The manuscript furthermore provides a pipeline for processing dynamic natural stimulus materials to allow for use in controlled research contexts.

## Supporting information

S1 Table All undertaken edits of the positive and negative facial expression stimuli divided into good and edited clips. Reference Clips refer to the video clips that were used as a reference for the appropriate peak time, but both the reference and the further clips had to be edited to fit the standardization criteria.
(PDF)

S2 Table Numbers of video clips in the two stimulus sets for each expression and elicitation method. (PDF)

S3 Table Mean ratings and *SD*s of each rating dimension of both study 1 and study 2 for positive and negative expression stimuli. In study 1 (*n* = 13) participants evaluated each stimulus. In study 2 (*n* = 68) each participant evaluated only half of the dynamic stimuli, so each dynamic stimulus was evaluated by 34 participants. “R” in the stimuli labels marks the reference stimuli with which the peak expression time was calculated.
(PDF)

S4 Table Mean ratings and *SD*s of each rating dimension of study 2 for neutral expression stimuli. The abbreviation “aP”in the video clip labels refers to the blinking starting after the peak expression (667ms). The abbreviation “bP” refers to the blinking starting before the peak expression.
(PDF)

S1 File Questions regarding the success of the elicitation conditions during the video recording. (PDF)

S2 File German explanations of the experimental flow and the rating scales. (PDF)

S3 File English explanations of the experimental flow and the rating scales. (PDF)

S4 File Assumption tests for the used statistical analyses. (PDF)

S5 File Detailed results of study 1: First onset frame validation. (PDF)
