# Supplementary material for: Development and validation of a natural dynamic facial expression stimulus set
Source: PLoS One. 2023 Jun 28;18(6):e0287049. doi: 10.1371/journal.pone.0287049 (PMC10306207; doi:10.1371/journal.pone.0287049)
Supplement: S1 Table — Reference Clips refer to the video clips that were used as a reference for the appropriate peak time, but both the reference and the further clips had to be edited to fit the standardization criteria. (PDF) [file pone.0287049.s001.pdf]

S1 Table. All Undertaken Edits of the Positive and Negative Facial Expression Stimuli Divided into Good and Edited Clips

Condition	Clip Category	Individual Clips	Frames Removed Overall	Frames Added Overall	Edits due to Blinking		Edits to Create Clear Onset		Edits to Match Peak Times	
					Removed	Added	Removed	Added	Removed	Added
Posed Positive										
	Reference Clips									
		P_pos_04_R	12	0	0	0	0	0	12	0
		P_pos_05_R	2	0	0	0	0	0	2	0
		P_pos_06_R	2	0	0	0	0	0	2	0
		P_pos_07_R	4	0	0	0	0	0	4	0
		P_pos_08_R	11	0	0	0	0	0	11	0
		P_pos_15_R	4	0	0	0	2	0	2	0
		P_pos_17_R	0	0	0	0	0	0	0	0
		P_pos_21_R	6	0	0	0	3	0	3	0
		P_pos_22_R	6	0	0	0	0	0	6	0
		P_pos_23_R	6	0	0	0	1	0	5	0
		P_pos_24_R	8	0	0	0	0	0	8	0
		P_pos_31_R	8	0	0	0	0	0	8	0
		P_pos_32_R	6	0	0	0	2	0	4	0
		P_pos_33_R	1	0	0	0	0	0	1	0
		P_pos_34_R	4	0	0	0	0	0	4	0
		P_pos_36_R	4	0	0	0	4	0	0	0
		P_pos_37_R	3	2	0	0	3	0	0	2
		P_pos_41_R	6	0	0	0	2	0	4	0
		P_pos_43_R	2	0	0	0	0	0	2	0
		P_pos_46_R	6	0	0	0	2	0	4	0
		P_pos_47_R	4	0	0	0	0	0	4	0
		P_pos_49_R	3	0	0	0	0	0	3	0
		P_pos_50_R	4	0	0	0	0	0	4	0
		P_pos_52_R	10	0	0	0	0	0	10	0

Condition	Clip Category	Individual Clips	Frames Removed Overall	Frames Added Overall	Edits due to Blinking		Edits to Create Clear Onset		Edits to Match Peak Times	
					Removed	Added	Removed	Added	Removed	Added

Condition	Clip Category	Individual Clips	Frames Removed Overall	Frames Added Overall	Edits due to Blinking		Edits to Create Clear Onset		Edits to Match Peak Times	
					Removed	Added	Removed	Added	Removed	Added
Posed Positive										
	Further Clips									
		P_pos_02	14	0	8	0	0	0	6	0
		P_pos_10	11	0	9	0	2	0	0	0
		P_pos_11	20	0	8	0	0	0	12	0
		P_pos_14	18	0	6	0	0	0	12	0
		P_pos_16	12	0	8	0	0	0	4	0
		P_pos_18	5	5	5	0	0	0	0	5
		P_pos_19	8	2	8	0	0	0	0	2
		P_pos_25	8	2	8	0	0	0	0	2
		P_pos_26	11	2	8	0	3	0	0	2
		P_pos_28	10	0	8	0	0	0	2	0
		P_pos_30	19	0	9	0	0	0	10	0
		P_pos_35	21	0	5	0	0	0	16	0
		P_pos_38	9	0	7	0	0	0	2	0
		P_pos_40	10	0	9	0	0	0	1	0
		P_pos_42	6	2	6	0	0	0	0	2
		P_pos_44	10	0	8	0	0	0	2	0
		P_pos_45	10	0	6	0	0	0	4	0
		P_pos_53	32	0	9	0	0	0	23	0
		P_pos_54	10	0	9	0	0	0	1	0
		P_pos_55	2	1	0	0	2	0	0	1
		P_pos_57	9	1	9	0	0	0	0	1
Posed Negative										
	Reference Clips									
		P_neg_01_R	6	0	0	0	0	0	6	0
		P_neg_02_R	0	7	0	0	0	0	0	7
		P_neg_08_R	1	0	0	0	0	0	1	0
		P_neg_09_R	14	0	0	0	10	0	4	0
		P_neg_10_R	0	0	0	0	0	0	0	0

Condition	Clip Category	Individual Clips	Frames Removed Overall	Frames Added Overall	Edits due to Blinking		Edits to Create Clear Onset		Edits to Match Peak Times	
					Removed	Added	Removed	Added	Removed	Added
Posed Negative										
	Reference Clips									
		P_neg_19_R	0	0	0	0	0	0	0	0
		P_neg_28_R	0	9	0	0	0	0	0	9
		P_neg_29_R	8	0	0	0	7	0	1	0
		P_neg_30_R	6	2	0	0	6	0	0	2
		P_neg_31_R	1	0	0	0	0	0	1	0
		P_neg_38_R	0	4	0	0	0	0	0	4
		P_neg_39_R	0	2	0	0	0	0	0	2
		P_neg_42_R	0	5	0	0	0	0	0	5
		P_neg_46_R	0	0	0	0	0	0	0	0
		P_neg_47_R	0	1	0	0	0	0	0	1
		P_neg_50_R	8	0	0	0	0	0	8	0
		P_neg_52_R	0	3	0	0	0	0	0	3
		P_neg_53_R	0	4	0	0	0	0	0	4
		P_neg_54_R	0	1	0	0	0	0	0	1
		P_neg_55_R	0	2	0	0	0	0	0	2
	Further Clips									
		P_neg_03	7	3	7	0	0	0	0	3
		P_neg_04	26	2	12	0	14	0	0	2
		P_neg_05	9	0	8	0	0	0	2	0
		P_neg_06	10	0	8	0	0	0	1	0
		P_neg_07	8	12	8	0	0	0	0	12
		P_neg_11	14	0	12	0	0	0	2	0
		P_neg_12	15	5	10	0	0	0	0	5
		P_neg_13	5	3	5	0	0	0	0	3
		P_neg_14	30	4	12	0	18	0	0	4
		P_neg_15	16	0	10	0	0	0	6	0
		P_neg_16	6	10	6	0	0	0	0	10
		P_neg_17	10	8	10	0	0	0	0	8
		P_neg_20	7	2	7	0	0	0	0	2

Condition	Clip Category	Individual Clips	Frames Removed Overall	Frames Added Overall	Edits due to Blinking		Edits to Create Clear Onset		Edits to Match Peak Times	
					Removed	Added	Removed	Added	Removed	Added
Posed Negative										
	Further Clips									
		P_neg_21	3	0	0	0	1	0	2	0
		P_neg_22	6	0	0	0	2	0	4	0
		P_neg_24	6	0	0	0	5	0	1	0
		P_neg_25	8	4	8	0	0	0	0	4
		P_neg_26	19	0	8	0	0	0	11	0
		P_neg_27	8	0	0	0	3	0	5	0
		P_neg_32	8	8	8	0	0	0	0	8
		P_neg_33	8	0	8	0	0	0	0	0
		P_neg_34	10	0	8	0	0	0	2	0
		P_neg_35	6	0	5	0	0	0	1	0
		P_neg_36	10	0	10	0	0	0	0	0
		P_neg_37	7	0	6	0	0	0	1	0
		P_neg_40	16	0	10	0	0	0	6	0
		P_neg_41	8	0	0	0	5	0	3	0
		P_neg_43	8	0	8	0	0	0	1	0
		P_neg_44	10	4	10	0	0	0	0	4
		P_neg_45	4	2	0	0	4	0	0	2
		P_neg_48	6	10	6	0	0	0	0	10
		P_neg_49	21	0	15	0	0	0	6	0
		P_neg_51	11	0	8	0	1	0	3	0
		P_neg_56	12	0	10	0	0	0	2	0
		P_neg_57	10	0	8	0	0	0	2	0
Event-Elicited Positive										
	Reference Clips									
		EE_pos_12_R	0	0	0	0	0	0	0	0
		EE_pos_14_R	0	0	0	0	0	0	0	0
		EE_pos_21_R	8	0	0	0	2	0	6	0
		EE_pos_25_R	1	0	0	0	0	0	1	0

Event-Elicited Positive										
	Reference Clips									
		EE_pos_35_R	11	0	0	0	3	0	8	0
		EE_pos_39_R	7	0	0	0	7	0	0	0
		EE_pos_45_R	6	0	0	0	0	0	6	0
		EE_pos_48_R	2	2	0	0	2	0	0	2
		EE_pos_50_R	3	0	0	0	0	0	3	0
		EE_pos_51_R	14	0	0	0	13	0	1	0
		EE_pos_57_R	2	0	0	0	0	0	2	0
	Further Clips									
		EE_pos_01	8	4	8	0	0	0	0	4
		EE_pos_04	8	0	6	0	0	0	2	0
		EE_pos_05	10	0	8	0	0	0	2	0
		EE_pos_06	8	0	8	0	0	0	0	0
		EE_pos_08	15	0	9	0	0	0	6	0
		EE_pos_09	6	5	6	0	0	0	0	5
		EE_pos_10	10	2	10	0	0	0	0	2
		EE_pos_16	8	0	8	0	0	0	0	0
		EE_pos_18	10	0	8	0	0	0	2	0
		EE_pos_26	8	2	8	0	0	0	0	2
		EE_pos_27	8	0	6	0	0	0	2	0
		EE_pos_28	12	0	8	0	0	0	4	0
		EE_pos_29	9	1	9	0	0	0	0	1
		EE_pos_31	18	0	0	0	16	0	2	0
		EE_pos_32	8	6	8	0	0	0	0	6
		EE_pos_33	6	2	6	0	0	0	0	2
		EE_pos_34	8	0	8	0	0	0	0	0
		EE_pos_36	8	0	6	0	0	0	2	0
		EE_pos_37	8	0	8	0	0	0	0	0
		EE_pos_38	1	0	8	0	0	0	6	0
Condition	Clip Category	Individual Clips	Frames Removed Overall	Frames Added Overall	Edits due to Blinking		Edits to Create Clear Onset		Edits to Match Peak Times	
					Removed	Added	Removed	Added	Removed	Added

Event-Elicited Positive										
	Further Clips									
		EE_pos_40	6	2	6	0	0	0	0	2
		EE_pos_41	22	0	7	0	4	0	11	0
		EE_pos_43	19	0	0	0	11	0	8	0
		EE_pos_44	16	0	8	0	0	0	8	0
		EE_pos_46	14	0	8	0	0	0	6	0
		EE_pos_47	12	0	7	0	0	0	5	0
		EE_pos_49	10	0	5	0	0	0	5	0
		EE_pos_52	6	4	6	0	0	0	0	4
		EE_pos_54	6	0	6	0	0	0	6	0
Event-Elicited Negative										
	Reference Clips									
		EE_neg_09_R	0	4	0	0	0	0	0	4
		EE_neg_10_R	0	0	0	0	0	0	0	0
		EE_neg_16_R	0	2	0	0	0	0	0	2
		EE_neg_18_R	0	2	0	0	0	0	0	2
		EE_neg_21_R	0	4	0	0	0	0	0	4
		EE_neg_27_R	0	3	0	0	0	0	0	3
		EE_neg_36_R	0	4	0	0	0	0	0	4
		EE_neg_39_R	0	4	0	0	0	0	0	4
		EE_neg_41_R	0	2	0	0	0	0	0	2
		EE_neg_42_R	0	4	0	0	0	0	0	4
		EE_neg_43_R	0	4	0	0	0	0	0	4
		EE_neg_49_R	0	5	0	0	0	0	0	5
		EE_neg_51_R	0	8	0	0	0	0	0	8
		EE_neg_55_R	0	8	0	0	0	0	0	8
		EE_neg_56_R	0	2	0	0	0	0	0	2
Condition	Clip Category	Individual Clips	Frames Removed Overall	Frames Added Overall	Edits due to Blinking		Edits to Create Clear Onset		Edits to Match Peak Times	
					Removed	Added	Removed	Added	Removed	Added

Event-Elicited Negative										
	Further Clips									
		EE_neg_02	6	10	5	0	1	0	0	10
		EE_neg_03	12	0	10	0	0	0	2	0
		EE_neg_04	5	0	5	0	0	0	0	0
		EE_neg_05	5	6	5	0	0	0	0	6
		EE_neg_06	6	10	6	0	0	0	0	10
		EE_neg_07	8	5	8	0	0	0	0	5
		EE_neg_11	10	2	10	0	0	0	0	2
		EE_neg_12	8	5	8	0	0	0	0	5
		EE_neg_13	8	4	8	0	0	0	0	4
		EE_neg_14	14	0	8	0	0	0	6	0
		EE_neg_15	8	0	0	0	5	0	3	0
		EE_neg_17	7	0	7	0	0	0	0	0
		EE_neg_19	10	0	8	0	0	0	2	0
		EE_neg_20	8	0	0	0	6	0	2	0
		EE_neg_22	11	0	9	0	0	0	2	0
		EE_neg_23	2	0	0	0	0	0	2	0
		EE_neg_24	6	2	6	0	0	0	0	2
		EE_neg_25	6	0	0	0	5	0	1	0
		EE_neg_28	15	3	11	0	4	0	0	3
		EE_neg_31	8	3	8	0	0	0	0	3
		EE_neg_32	6	0	0	0	5	0	1	0
		EE_neg_33	8	2	8	0	0	0	0	2
		EE_neg_35	10	0	9	0	0	0	1	0
		EE_neg_40	6	2	6	0	0	0	0	2
		EE_neg_45	8	0	7	0	0	0	1	0
		EE_neg_46	10	0	8	0	0	0	2	0
		EE_neg_47	0	1	0	0	0	0	0	1
		EE_neg_48	6	4	6	0	0	0	0	4
		EE_neg_50	10	0	8	0	0	0	2	0
Condition	Clip Category	Individual Clips	Frames Removed Overall	Frames Added Overall	Edits due to Blinking		Edits to Create Clear Onset		Edits to Match Peak Times	
					Removed	Added	Removed	Added	Removed	Added

Event-Elicited Negative									
Further Clips									
	EE_neg_53	6	2	6	0	0	0	0	2
	EE_neg_54	2	10	0	0	2	0	0	10
	EE_neg_57	6	2	6	0	0	0	0	2

Note. Reference Clips refer to the video clips that were used as a reference for the appropriate peak time, but both the reference and the further clips had to be edited to fit the standardization criteria
