# Supplementary material for: Development and validation of a natural dynamic facial expression stimulus set
Source: PLoS One. 2023 Jun 28;18(6):e0287049. doi: 10.1371/journal.pone.0287049 (PMC10306207; doi:10.1371/journal.pone.0287049)
Supplement: S2 Table — (PDF) [file pone.0287049.s002.pdf]

S2 Table. Numbers of Video Clips in the Two Stimulus Sets for Each Expression and Elicitation Method.

Stimulus Set	Elicitation Method	Neutral Expression	Positive Expression	Negative Expression
Set 1				
	Event-Elicited	28	19	23
	Posed	28	22	27
Set 2				
	Event-Elicited	27	18	22
	Posed	29	23	28
