# Supplementary material for: Development and validation of a natural dynamic facial expression stimulus set
Source: PLoS One. 2023 Jun 28;18(6):e0287049. doi: 10.1371/journal.pone.0287049 (PMC10306207; doi:10.1371/journal.pone.0287049)
Supplement: S3 Table — In study 1 (n = 13) participants evaluated each stimulus. In study 2 (n = 68) each participant evaluated only half of the dynamic stimuli, so each dynamic stimulus was evaluated by 34 participants. “R” in the stimuli labels marks the reference stimuli with which the peak expression time was calculated. (PDF) [file pone.0287049.s003.pdf]

S3 Table. Mean Ratings and SDs of each Rating Dimension of Both Study 1 and Study 2 for Positive and Negative Expression Stimuli

Condition	Elicitation Method	Individual Clips	Study 1: First Onset Frame Validation				Study 2: Dynamic Stimuli Validation							
			Valence		Intensity		Valence		Intensity		Genuineness		Perceived Edits	
			M	*(SD)*	*M*	*(SD)*	*M*	*(SD)*	*M*	*(SD)*	*M*	*(SD)*	*M*	*(SD)*
Positive														
	Posed													
		P_pos_02	4.54	(1.56)	5.77	(1.59)	0.00	(3.24)	4.09	(2.82)	1.53	(3.69)	1.88	(0.33)
		P_pos_10	4.23	(1.59)	6.15	(1.52)	0.71	(3.17)	3.41	(2.76)	2.38	(2.81)	1.97	(0.17)
		P_pos_11	1.92	(1.85)	4.00	(1.63)	1.29	(3.58)	4.24	(2.52)	2.35	(3.84)	1.85	(0.36)
		P_pos_14	4.31	(1.93)	5.92	(2.40)	0.59	(3.45)	3.79	(2.82)	1.41	(3.69)	1.82	(0.39)
		P_pos_16	1.62	(1.26)	3.92	(1.85)	1.47	(3.05)	4.26	(2.71)	0.74	(3.82)	1.76	(0.43)
		P_pos_18	2.31	(1.84)	4.23	(2.13)	0.26	(3.42)	4.21	(2.63)	2.41	(3.04)	1.88	(0.33)
		P_pos_19	2.62	(2.14)	4.62	(1.56)	1.38	(2.98)	3.97	(2.76)	2.24	(3.63)	1.85	(0.36)
		P_pos_25	1.54	(1.45)	4.46	(1.27)	1.56	(3.40)	4.26	(2.83)	2.09	(3.48)	1.79	(0.41)
		P_pos_26	1.69	(2.18)	4.31	(1.89)	1.56	(3.21)	3.79	(3.10)	2.56	(3.74)	1.88	(0.33)
		P_pos_28	2.38	(2.10)	4.15	(2.23)	0.94	(3.44)	3.59	(2.90)	2.06	(3.72)	1.91	(0.29)
		P_pos_30	1.77	(1.42)	4.54	(1.76)	0.50	(3.49)	4.53	(2.11)	1.50	(3.36)	1.88	(0.33)
		P_pos_35	1.62	(1.45)	3.92	(1.89)	1.53	(3.08)	4.41	(2.83)	0.71	(4.37)	1.76	(0.43)
		P_pos_38	3.77	(2.49)	5.54	(1.66)	0.71	(3.33)	4.32	(2.93)	0.62	(3.83)	1.88	(0.33)
		P_pos_40	0.77	(1.09)	3.38	(2.06)	2.35	(2.85)	3.94	(3.26)	3.00	(3.12)	1.91	(0.29)
		P_pos_42	4.46	(1.66)	6.31	(1.70)	0.91	(2.77)	3.44	(2.43)	1.38	(3.39)	1.82	(0.39)
		P_pos_44	4.31	(1.60)	6.00	(1.47)	0.09	(2.42)	2.97	(2.21)	0.79	(3.06)	1.76	(0.43)
		P_pos_45	0.62	(1.39)	4.08	(1.55)	0.82	(3.02)	4.15	(2.76)	2.62	(2.71)	1.88	(0.33)
		P_pos_53	0.85	(2.30)	3.85	(2.34)	0.50	(3.61)	4.09	(2.26)	1.82	(4.04)	1.85	(0.36)
		P_pos_54	4.31	(2.25)	6.08	(1.71)	0.68	(3.23)	4.65	(2.21)	0.74	(3.65)	1.79	(0.41)
		P_pos_55	2.46	(1.20)	4.62	(1.80)	0.29	(3.49)	3.71	(2.67)	2.35	(4.30)	1.79	(0.41)
		P_pos_57	1.46	(1.20)	3.85	(1.63)	1.03	(3.10)	3.76	(2.87)	1.97	(3.75)	1.91	(0.29)
		P_pos_04_R	2.31	(1.25)	4.23	(1.96)	0.50	(3.38)	3.62	(2.70)	1.41	(4.01)	1.94	(0.24)
		P_pos_05_R	3.00	(1.68)	5.31	(1.70)	1.47	(2.58)	3.68	(3.19)	2.00	(3.44)	1.88	(0.33)
		P_pos_06_R	2.23	(1.30)	4.46	(1.51)	0.97	(3.66)	4.21	(2.77)	2.06	(3.27)	1.91	(0.29)
		P_pos_07_R	2.62	(1.33)	4.46	(1.56)	2.06	(3.34)	4.68	(2.67)	3.00	(3.17)	2.00	(0.00)

Condition	Elicitation Method	Individual Clips	Study 1: First Onset Frame Validation				Study 2: Dynamic Stimuli Validation							
			Valence		Intensity		Valence		Intensity		Genuineness		Perceived Edits	
			M	*(SD)*	*M*	*(SD)*	*M*	*(SD)*	*M*	*(SD)*	*M*	*(SD)*	*M*	*(SD)*
Positive														
	Posed													
		P_pos_08_R	3.31	(1.70)	5.54	(1.66)	0.88	(3.59)	4.03	(2.79)	1.56	(4.14)	1.94	(0.24)
		P_pos_15_R	2.69	(1.32)	4.69	(2.32)	1.38	(3.46)	4.56	(2.72)	1.59	(3.93)	1.79	(0.41)
		P_pos_17_R	2.54	(2.15)	4.69	(1.84)	0.68	(2.91)	3.79	(2.35)	1.35	(3.83)	1.85	(0.36)
		P_pos_21_R	1.15	(1.21)	4.23	(2.01)	1.91	(3.18	4.12	(2.75)	1.76	(3.81)	1.88	(0.33)
		P_pos_22_R	-0.08	(0.49)	3.38	(2.18)	0.03	(3.61)	3.76	(2.63)	2.24	(3.46)	1.85	(0.36)
		P_pos_23_R	0.15	(0.69)	3.23	(2.49)	0.62	(3.64)	4.59	(2.56)	1.41	(4.21)	1.91	(0.29)
		P_pos_24_R	1.23	(1.42)	4.00	(1.63)	-0.03	(3.15)	3.29	(2.29)	1.62	(4.12)	1.88	(0.33)
		P_pos_31_R	1.77	(1.69)	4.08	(1.85)	0.94	(3.05)	4.12	(2.77)	2.35	(3.02)	1.91	(0.29)
		P_pos_32_R	2.46	(1.66)	4.54	(1.94)	1.35	(3.24)	3.85	(2.88)	1.85	(3.77)	1.79	(0.41)
		P_pos_33_R	0.23	(0.83)	3.31	(2.14)	1.09	(3.00)	3.62	(2.35)	2.29	(3.39)	1.97	(0.17)
		P_pos_34_R	1.31	(0.85)	4.23	(1.42)	2.29	(3.48)	5.12	(2.43)	2.18	(3.19)	1.94	(0.24)
		P_pos_36_R	2.00	(1.00)	4.23	(1.83)	1.53	(3.02)	3.53	(2.75)	2.15	(3.88)	1.85	(0.36)
		P_pos_37_R	0.69	(1.11)	3.38	(1.80)	1.47	(3.43)	3.74	(2.87)	2.82	(3.25)	1.91	(0.29)
		P_pos_41_R	2.92	(1.61)	4.62	(1.80)	1.62	(3.58)	4.47	(2.55)	1.32	(3.91)	1.74	(0.45)
		P_pos_43_R	0.15	(1.28)	3.15	(1.95)	0.88	(3.34)	3.56	(3.02)	3.15	(3.38)	1.94	(0.24)
		P_pos_46_R	3.00	(1.83)	4.92	(1.75)	1.47	(3.23)	4.50	(2.69)	2.21	(3.69)	1.88	(0.33)
		P_pos_47_R	1.92	(1.19)	4.15	(1.72)	1.79	(3.73)	4.50	(2.71)	2.62	(3.19)	1.85	(0.36)
		P_pos_49_R	1.69	(1.89)	4.08	(2.06)	1.85	(3.31)	4.38	(2.91)	2.82	(3.72)	1.94	(0.24)
		P_pos_50_R	1.54	(0.78)	3.69	(1.44)	0.06	(3.26)	4.03	(2.38)	1.03	(3.29)	1.94	(0.24)
		P_pos_52_R	0.92	(0.64)	3.08	(1.50)	0.56	(2.84)	3.65	(2.79)	2.26	(3.15)	1.91	(0.29)
	Event-Elicited													
		EE_pos_01	4.15	(1.72)	6.00	(1.91)	0.68	(3.53)	3.94	(3.29)	1.44	(3.59)	1.94	(0.24)
		EE_pos_04	3.15	(1.82)	4.85	(1.82)	0.44	(3.04)	3.44	(3.15)	1.35	(4.16)	1.82	(0.39)
		EE_pos_05	6.23	(0.83)	7.69	(0.95)	0.65	(3.79)	4.26	(3.03)	1.09	(4.00)	1.91	(0.29)
		EE_pos_06	3.69	(1.65)	5.46	(1.56)	1.53	(3.76)	5.18	(2.74)	1.82	(3.75)	1.91	(0.29)
		EE_pos_08	1.23	(1.42)	3.46	(1.98)	1.38	(3.61)	4.32	(2.93)	1.41	(4.35)	1.91	(0.29)
		EE_pos_09	1.77	(1.42)	4.46	(1.33)	0.44	(3.51)	4.53	(3.12)	0.35	(4.13)	1.88	(0.33)

Condition	Elicitation Method	Individual Clips	Study 1: First Onset Frame Validation				Study 2: Dynamic Stimuli Validation							
			Valence		Intensity		Valence		Intensity		Genuineness		Perceived Edits	
			M	*(SD)*	*M*	*(SD)*	*M*	*(SD)*	*M*	*(SD)*	*M*	*(SD)*	*M*	*(SD)*
Positive														
	Event-Elicited													
		EE_pos_10	1.85	(1.41)	4.15	(2.03)	-0.06	(3.27)	4.15	(2.95)	0.97	(4.46)	1.79	(0.41)
		EE_pos_16	4.15	(1.52)	6.00	(1.68)	0.35	(3.37)	4.35	(2.48)	1.35	(3.89)	1.82	(0.39)
		EE_pos_18	3.92	(1.71)	6.08	(1.80)	0.35	(3.22)	4.03	(2.68)	1.24	(4.47)	1.88	(0.33)
		EE_pos_26	3.62	(2.43)	5.85	(1.77)	1.47	(3.58)	4.32	(2.64)	-0.15	(4.39)	1.71	(0.46)
		EE_pos_28	1.92	(2.56)	4.38	(2.06)	-0.09	(2.47)	3.32	(2.36)	1.18	(3.54)	1.82	(0.39)
		EE_pos_29	3.92	(2.33)	6.00	(1.68)	0.12	(2.83)	3.97	(2.63)	0.62	(4.13)	1.91	(0.29)
		EE_pos_31	2.23	(1.54)	4.31	(1.84)	1.38	(3.65)	4.50	(2.83)	2.15	(4.21)	1.88	(0.33)
		EE_pos_32	4.62	(1.39)	6.62	(1.39)	0.76	(3.50)	4.26	(2.61)	0.79	(4.38)	1.85	(0.36)
		EE_pos_33	2.38	(2.43)	5.38	(1.89)	0.65	(3.10)	4.12	(2.82)	1.35	(3.84)	1.82	(0.39)
		EE_pos_34	4.15	(1.72)	5.69	(1.65)	0.47	(3.11)	4.03	(2.72)	0.62	(3.52)	1.82	(0.39)
		EE_pos_36	3.00	(2.12)	5.00	(1.87)	0.56	(3.13)	3.74	(2.54)	2.44	(4.00)	1.91	(0.29)
		EE_pos_37	5.08	(1.26)	6.46	(1.81)	1.38	(3.36)	3.97	(2.76)	0.41	(4.16)	1.91	(0.29)
		EE_pos_38	0.85	(2.58)	4.77	(1.74)	0.24	(3.04)	3.56	(3.14)	1.71	(3.99)	1.85	(0.36)
		EE_pos_40	4.08	(1.50)	5.92	(1.66)	2.12	(3.46)	4.85	(2.93)	1.03	(4.14)	1.76	(0.43)
		EE_pos_41	0.31	(2.39)	4.54	(1.27)	0.76	(3.37)	4.32	(2.92)	2.09	(4.01)	1.85	(0.36)
		EE_pos_43	1.62	(1.04)	3.62	(1.71)	0.65	(3.45)	4.15	(2.55)	1.21	(3.57)	1.91	(0.29)
		EE_pos_44	4.23	(1.74)	6.31	(2.14)	0.18	(3.49)	3.85	(2.82)	2.47	(4.14)	1.94	(0.24)
		EE_pos_46	3.15	(2.51)	5.15	(2.19)	1.35	(3.53)	4.38	(3.07)	0.79	(4.14)	1.91	(0.29)
		EE_pos_52	4.69	(1.65)	6.54	(1.45)	0.85	(3.45)	3.88	(3.01)	3.15	(3.60)	2.00	(0.00)
		EE_pos_54	1.31	(2.21)	3.77	(1.79)	0.82	(3.63)	4.18	(2.81)	1.24	(3.84)	1.97	(0.17)
		EE_pos_12_R	1.38	(1.45)	3.92	(2.14)	0.74	(3.16)	4.15	(2.91)	0.88	(4.21)	1.91	(0.29)
		EE_pos_14_R	1.08	(1.12)	3.92	(1.55)	0.91	(3.63)	4.56	(2.78)	-0.21	(3.91)	1.88	(0.33)
		EE_pos_21_R	2.46	(1.39)	4.46	(1.71)	0.35	(3.36)	3.85	(2.61)	1.32	(3.63)	1.91	(0.29)
		EE_pos_25_R	1.31	(0.95)	3.69	(1.18)	0.94	(2.73)	4.03	(2.60)	1.06	(3.17)	1.94	(0.24)
		EE_pos_35_R	0.38	(0.65)	3.15	(1.95)	1.06	(3.63)	4.47	(2.90)	0.47	(4.34)	1.85	(0.36)
		EE_pos_39_R	2.85	(1.91)	4.85	(2.48)	0.97	(3.57)	4.06	(2.82)	2.24	(3.57)	1.91	(0.29)
		EE_pos_45_R	2.31	(1.75)	4.08	(2.25)	2.03	(3.30)	4.41	(2.83)	2.97	(3.60)	1.85	(0.36)
		EE_pos_48_R	2.46	(1.51)	4.46	(1.90)	0.59	(3.72)	5.09	(2.76)	1.91	(3.82)	1.91	(0.29)

Condition	Elicitation Method	Individual Clips	Study 1: First Onset Frame Validation				Study 2: Dynamic Stimuli Validation							
			Valence		Intensity		Valence		Intensity		Genuineness		Perceived Edits	
			M	*(SD)*	*M*	*(SD)*	*M*	*(SD)*	*M*	*(SD)*	*M*	*(SD)*	*M*	*(SD)*
Positive														
	Event-Elicited													
		EE_pos_50_R	0.77	(1.88)	4.23	(1.59)	1.12	(3.09)	4.15	(2.98)	2.00	(4.08)	1.82	(0.39)
		EE_pos_51_R	1.23	(1.36)	3.69	(2.10)	1.50	(3.37)	4.47	(2.81)	1.06	(3.90)	1.74	(0.45)
		EE_pos_57_R	1.23	(1.79)	4.08	(2.14)	1.53	(3.61)	4.56	(3.07)	0.71	(3.76)	1.85	(0.36)
Negative														
	Posed													
		P_neg_03	-1.77	(1.59)	3.69	(1.75)	-0.53	(3.32)	3.71	(2.97)	1.03	(3.65)	1.91	(0.29)
		P_neg_04	-0.69	(0.85)	3.08	(1.85)	-0.53	(3.44)	3.71	(3.05)	2.21	(3.86)	1.88	(0.33)
		P_neg_05	-2.77	(1.74)	4.15	(2.15)	-0.38	(2.55)	2.97	(2.71)	1.74	(3.74)	1.85	(0.36)
		P_neg_06	-0.46	(1.27)	2.77	(1.64)	-0.06	(3.61)	4.00	(2.46)	1.03	(4.10)	1.76	(0.43)
		P_neg_07	-2.38	(1.33)	4.62	(2.10)	-0.32	(3.41)	4.00	(2.55)	2.09	(3.61)	1.85	(0.36)
		P_neg_11	-2.85	(1.57)	4.77	(1.48)	-0.38	(2.73)	2.79	(2.58)	2.35	(2.85)	1.91	(0.29)
		P_neg_12	-2.62	(1.45)	4.54	(2.26)	0.50	(2.98)	3.82	(2.80)	1.94	(3.36)	1.91	(0.29)
		P_neg_13	-2.31	(1.55)	4.31	(1.93)	-0.38	(3.22)	3.24	(2.09)	2.21	(3.48)	1.85	(0.36)
		P_neg_14	-1.15	(2.03)	3.08	(1.89)	-1.06	(2.44)	3.74	(2.38)	1.85	(3.33)	1.91	(0.29)
		P_neg_15	-1.85	(1.72)	3.62	(2.36)	-1.00	(3.16)	4.09	(2.44)	1.74	(3.72)	1.82	(0.39)
		P_neg_16	-2.31	(1.32)	4.00	(1.96)	0.74	(3.42)	4.47	(2.38)	2.21	(3.08)	1.88	(0.33)
		P_neg_17	-3.62	(1.19)	5.00	(1.68)	-0.03	(3.75)	4.15	(2.38)	1.91	(3.09)	1.88	(0.33)
		P_neg_20	-1.92	(2.18)	4.77	(1.88)	-0.71	(2.70)	3.15	(2.32)	1.82	(3.49)	1.74	(0.45)
		P_neg_21	-0.08	(0.64)	2.62	(1.98)	-0.15	(2.54)	3.21	(2.64)	2.53	(2.81)	1.82	(0.39)
		P_neg_22	-0.15	(1.41)	2.92	(1.75)	-0.06	(3.19)	3.91	(2.73)	2.47	(3.37)	1.88	(0.33)
		P_neg_24	-0.31	(1.60)	3.46	(1.39)	-0.59	(2.81)	3.35	(2.41)	2.15	(3.04)	1.94	(0.24)
		P_neg_25	-3.54	(1.98)	5.62	(1.66)	-0.06	(3.47)	3.91	(2.92)	2.24	(2.71)	1.91	(0.29)
		P_neg_26	-2.54	(1.76)	4.77	(2.01)	-0.71	(2.39)	3.18	(2.59)	2.09	(3.24)	1.94	(0.24)
		P_neg_27	-0.85	(0.99)	3.38	(2.26)	0.15	(2.82)	3.50	(2.84)	1.53	(3.44)	1.91	(0.29)
		P_neg_32	-2.85	(2.41)	5.15	(1.68)	-0.26	(2.76)	3.15	(2.26)	2.15	(3.55)	1.85	(0.36)
		P_neg_33	-2.46	(1.33)	4.31	(1.75)	-1.15	(2.69)	3.71	(2.42)	0.94	(3.08)	1.97	(0.17)
		P_neg_34	-3.69	(1.38)	5.54	(2.18)	-0.44	(3.08)	3.79	(2.64)	2.15	(3.79)	1.79	(0.41)

Condition	Elicitation Method	Individual Clips	Study 1: First Onset Frame Validation				Study 2: Dynamic Stimuli Validation							
			Valence		Intensity		Valence		Intensity		Genuineness		Perceived Edits	
			M	*(SD)*	*M*	*(SD)*	*M*	*(SD)*	*M*	*(SD)*	*M*	*(SD)*	*M*	*(SD)*
Negative														
	Posed													
		P_neg_35	-0.77	(1.42)	2.92	(2.40)	-0.21	(3.62)	3.82	(2.37)	1.29	(3.87)	1.82	(0.39)
		P_neg_36	-3.31	(1.32)	4.77	(1.74)	-0.68	(3.01)	3.74	(2.73)	1.79	(2.75)	1.91	(0.29)
		P_neg_37	-1.85	(1.52)	3.85	(2.15)	-0.38	(2.74)	3.65	(2.64)	2.03	(3.60)	1.91	(0.29)
		P_neg_40	-2.08	(1.19)	3.62	(1.45)	-1.12	(2.21)	3.15	(2.19)	2.82	(2.49)	1.97	(0.17)
		P_neg_41	-0.54	(1.90)	3.31	(1.93)	-1.65	(2.33)	3.41	(2.63)	2.38	(3.42)	1.91	(0.29)
		P_neg_43	-2.23	(1.36)	4.15	(1.82)	-1.35	(2.59)	3.47	(2.74)	2.06	(3.14)	1.88	(0.33)
		P_neg_44	-3.15	(1.52)	4.92	(1.55)	-1.15	(2.68)	4.15	(2.26)	2.09	(2.99)	1.85	(0.36)
		P_neg_45	-0.46	(0.88)	3.62	(1.71)	0.03	(3.24)	3.74	(2.55)	2.29	(3.72)	1.85	(0.36)
		P_neg_48	-3.69	(1.60)	5.77	(1.30)	-0.65	(2.55)	3.26	(2.60)	2.15	(3.60)	1.91	(0.29)
		P_neg_49	-0.69	(0.85)	3.23	(1.79)	-0.53	(3.01)	4.03	(2.74)	2.35	(3.72)	1.82	(0.39)
		P_neg_51	-1.31	(1.60)	3.62	(2.10)	-0.24	(2.95)	3.41	(2.63)	2.12	(3.65)	1.94	(0.24)
		P_neg_56	-2.00	(1.47)	4.08	(1.55)	0.03	(3.33)	4.15	(2.63)	2.68	(2.88)	1.88	(0.33)
		P_neg_57	-3.08	(2.25)	5.23	(1.48)	-0.06	(3.04)	3.59	(2.49)	2.59	(2.92)	1.88	(0.33)
		P_neg_01_R	-0.62	(1.26)	3.23	(1.88)	-0.06	(3.26)	4.35	(2.52)	2.41	(3.39)	1.94	(0.24)
		P_neg_02_R	-0.54	(1.85)	3.31	(2.06)	-1.18	(2.60)	3.65	(2.66)	0.74	(4.11)	1.85	(0.36)
		P_neg_08_R	-0.69	(0.95)	2.85	(1.72)	0.06	(3.44)	3.97	(2.71)	1.91	(3.52)	1.76	(0.43)
		P_neg_09_R	-0.62	(1.12)	3.23	(2.09)	-0.94	(3.13)	3.76	(2.67)	2.41	(3.12)	1.91	(0.29)
		P_neg_10_R	-1.00	(1.35)	2.92	(2.06)	-0.50	(2.65)	3.38	(2.94)	1.79	(3.30)	1.88	(0.33)
		P_neg_19_R	0.31	(0.95)	3.46	(1.94)	-0.41	(2.84)	3.85	(2.83)	1.26	(3.89)	1.79	(0.41)
		P_neg_28_R	-0.54	(0.78)	3.23	(1.74)	-1.59	(2.58)	3.41	(2.43)	1.71	(3.16)	1.91	(0.29)
		P_neg_29_R	-1.46	(1.20)	3.69	(2.02)	-0.35	(2.68)	3.88	(2.51)	2.35	(3.76)	1.91	(0.29)
		P_neg_30_R	-1.31	(1.25)	3.08	(2.29)	-1.21	(3.17)	4.47	(2.95)	2.06	(3.11)	1.85	(0.36)
		P_neg_31_R	0.23	(0.60)	3.23	(1.69)	-0.09	(2.81)	4.35	(2.93)	2.50	(3.33)	1.91	(0.29)
		P_neg_38_R	-1.23	(1.01)	3.77	(1.74)	-0.65	(3.63)	4.26	(2.50)	2.59	(3.52)	1.85	(0.36)
		P_neg_39_R	0.15	(0.55)	3.31	(2.29)	-0.38	(2.77)	3.41	(2.51)	1.68	(3.24)	1.94	(0.24)
		P_neg_42_R	-0.46	(0.66)	3.08	(2.18)	0.03	(3.38)	4.15	(2.55)	1.00	(3.81)	1.85	(0.36)
		P_neg_46_R	-1.08	(0.86)	3.15	(1.82)	0.03	(2.82)	3.65	(2.39)	2.21	(3.57)	1.88	(0.33)
		P_neg_47_R	-0.69	(0.75)	3.54	(1.81)	-1.00	(2.90)	3.71	(2.47)	2.97	(3.25)	1.91	(0.29)
		P_neg_50_R	-0.92	(1.19)	3.85	(2.12)	-0.74	(3.29)	4.21	(2.64)	2.88	(3.10)	1.94	(0.24)
		P_neg_52_R	-1.08	(1.19)	2.77	(2.20)	-0.47	(3.36)	3.41	(2.70)	2.24	(3.65)	1.85	(0.36)

Condition	Elicitation Method	Individual Clips	Study 1: First Onset Frame Validation				Study 2: Dynamic Stimuli Validation							
			Valence		Intensity		Valence		Intensity		Genuineness		Perceived Edits	
			M	*(SD)*	*M*	*(SD)*	*M*	*(SD)*	*M*	*(SD)*	*M*	*(SD)*	*M*	*(SD)*
Negative														
	Posed													
		P_neg_53_R	-1.23	(0.93)	3.23	(2.01)	0.41	(3.20)	3.97	(2.52)	1.12	(4.21)	1.88	(0.33)
		P_neg_54_R	-0.69	(1.11)	2.85	(1.99)	-1.35	(2.62)	3.38	(2.39)	1.88	(3.37)	1.88	(0.33)
		P_neg_55_R	-0.92	(1.19)	3.46	(2.07)	-1.35	(3.09)	3.91	(2.22)	0.91	(3.60)	1.88	(0.33)
	Event-Elicited													
		EE_neg_02	-0.08	(0.64)	3.46	(1.90)	0.76	(3.38)	3.68	(2.86)	2.12	(3.57)	1.82	(0.39)
		EE_neg_04	-2.92	(1.80)	4.77	(1.83)	0.06	(3.27)	3.50	(2.53)	1.82	(3.01)	1.91	(0.29)
		EE_neg_05	-2.69	(1.18)	4.92	(1.75)	-0.53	(3.11)	3.50	(2.45)	1.56	(3.36)	1.76	(0.43)
		EE_neg_06	-3.46	(1.45)	5.00	(1.91)	0.50	(3.50)	4.38	(2.91)	2.59	(3.72)	1.88	(0.33)
		EE_neg_11	-2.54	(1.51)	4.46	(1.71)	-0.53	(3.31)	3.53	(2.68)	1.56	(2.94)	1.79	(0.41)
		EE_neg_12	-2.38	(1.56)	4.31	(1.93)	0.76	(2.45)	3.59	(2.99)	2.03	(3.59)	1.85	(0.36)
		EE_neg_13	-3.23	(1.48)	5.31	(1.75)	-1.56	(2.65)	2.82	(2.02)	2.88	(2.51)	1.88	(0.33)
		EE_neg_14	-1.92	(0.95)	4.38	(1.61)	0.24	(3.17)	3.68	(2.69)	0.91	(3.49)	1.82	(0.39)
		EE_neg_15	-1.15	(1.57)	3.00	(2.08)	-0.32	(3.06)	4.35	(2.45)	1.68	(3.53)	1.85	(0.36)
		EE_neg_17	-2.15	(1.63)	3.69	(1.65)	0.21	(2.63)	3.26	(2.34)	1.91	(3.42)	1.85	(0.36)
		EE_neg_19	-2.69	(1.32)	5.00	(1.78)	-0.76	(2.95)	3.62	(3.06)	2.29	(3.27)	1.88	(0.33)
		EE_neg_20	-0.08	(0.95)	3.15	(1.72)	-0.53	(3.12)	3.76	(2.72)	2.12	(3.68)	1.97	(0.17)
		EE_neg_22	-3.46	(1.56)	5.23	(2.01)	-0.65	(2.67)	3.44	(2.67)	1.97	(3.24)	1.79	(0.41)
		EE_neg_23	-3.62	(1.66)	5.38	(1.89)	-0.82	(2.53)	3.00	(2.50)	2.21	(3.27)	1.91	(0.29)
		EE_neg_24	-2.69	(1.75)	4.38	(2.02)	-0.62	(3.07)	3.24	(2.43)	1.68	(2.92)	1.85	(0.36)
		EE_neg_25	-0.15	(0.55)	3.00	(1.58)	-0.38	(3.41)	4.35	(2.46)	2.12	(3.26)	1.85	(0.36)
		EE_neg_28	-0.69	(1.03)	3.23	(1.74)	-0.53	(3.15)	4.26	(2.43)	1.88	(3.68)	1.94	(0.24)
		EE_neg_31	-3.46	(2.40)	6.00	(1.68)	0.06	(3.72)	3.53	(2.98)	1.56	(4.11)	1.76	(0.43)
		EE_neg_32	0.69	(0.85)	3.46	(1.98)	-0.29	(2.83)	3.35	(2.50)	2.03	(3.41)	1.94	(0.24)
		EE_neg_33	-4.00	(1.68)	5.62	(1.50)	0.18	(3.46)	4.12	(2.57)	1.94	(3.74)	1.82	(0.39)
		EE_neg_35	-3.77	(1.96)	5.15	(2.15)	-0.44	(3.21)	3.85	(2.63)	2.06	(3.12)	1.82	(0.39)
		EE_neg_40	-3.77	(1.96)	5.08	(2.47)	-0.82	(2.19)	3.65	(2.50)	1.74	(3.31)	1.85	(0.36)
		EE_neg_45	-3.15	(1.46)	5.00	(1.96)	-0.18	(3.22)	3.97	(2.66)	1.94	(3.30)	1.82	(0.39)

Condition	Elicitation Method	Individual Clips	Study 1: First Onset Frame Validation				Study 2: Dynamic Stimuli Validation							
			Valence		Intensity		Valence		Intensity		Genuineness		Perceived Edits	
			M	*(SD)*	*M*	*(SD)*	*M*	*(SD)*	*M*	*(SD)*	*M*	*(SD)*	*M*	*(SD)*
Negative														
	Event-Elicited													
		EE_neg_46	-2.92	(1.44)	4.92	(1.89)	-0.76	(2.87)	4.03	(2.37)	2.00	(3.12)	1.94	(0.24)
		EE_neg_47	-1.23	(0.83)	3.31	(2.02)	-0.50	(3.65)	3.94	(2.76)	2.12	(3.56)	1.91	(0.29)
		EE_neg_48	-3.77	(1.64)	5.00	(2.31)	-1.00	(3.07)	4.09	(2.39)	2.24	(3.04)	1.94	(0.24)
		EE_neg_50	-4.77	(1.48)	6.15	(1.91)	-1.38	(2.56)	3.18	(2.42)	2.26	(3.66)	1.79	(0.41)
		EE_neg_53	-2.00	(1.78)	4.23	(1.54)	0.00	(2.82)	3.62	(2.67)	1.85	(3.29)	1.97	(0.17)
		EE_neg_54	-1.54	(1.33)	4.08	(2.36)	-0.50	(3.99)	4.29	(2.90)	2.26	(3.54)	1.85	(0.36)
		EE_neg_57	-3.23	(1.69)	4.46	(2.15)	0.32	(3.58)	3.94	(2.91)	1.97	(3.73)	1.88	(0.33)
		EE_neg_09_R	-0.38	(0.65)	2.85	(1.86)	-0.94	(3.28)	3.15	(2.86)	2.76	(3.42)	1.88	(0.33)
		EE_neg_10_R	0.15	(0.55)	2.85	(2.08)	-0.18	(3.21)	3.91	(2.64)	1.79	(3.29)	1.91	(0.29)
		EE_neg_16_R	-0.54	(0.78)	3.08	(1.89)	-0.35	(3.97)	4.53	(2.46)	2.76	(2.71)	1.88	(0.33)
		EE_neg_18_R	0.31	(0.85)	3.31	(2.18)	-0.97	(2.85)	3.76	(2.15)	2.32	(3.07)	1.91	(0.29)
		EE_neg_21_R	-0.54	(0.78)	3.31	(2.10)	-0.94	(2.97)	3.24	(2.57)	2.32	(3.55)	1.82	(0.39)
		EE_neg_27_R	-0.62	(1.12)	3.46	(2.37)	-0.65	(2.67)	3.68	(2.31)	1.94	(3.11)	1.94	(0.24)
		EE_neg_36_R	-1.08	(1.19)	3.46	(1.94)	-0.26	(3.02)	3.76	(2.45)	2.24	(3.23)	1.85	(0.36)
		EE_neg_39_R	-1.15	(1.68)	3.31	(2.29)	-0.06	(3.30)	3.79	(2.92)	2.15	(3.26)	1.88	(0.33)
		EE_neg_41_R	-1.23	(1.17)	2.85	(1.82)	-1.32	(2.91)	3.38	(2.50)	1.94	(3.27)	1.82	(0.39)
		EE_neg_42_R	-1.23	(1.48)	3.54	(2.11)	-0.68	(3.25)	3.88	(2.37)	1.32	(3.49)	1.85	(0.36)
		EE_neg_43_R	-0.85	(1.99)	3.23	(2.35)	-0.03	(2.69)	2.97	(2.53)	2.44	(3.08)	1.85	(0.36)
		EE_neg_49_R	-1.15	(1.21)	3.08	(1.71)	-0.50	(3.04)	3.56	(2.87)	2.38	(3.13)	1.94	(0.24)
		EE_neg_51_R	-1.85	(1.46)	4.00	(1.68)	-0.82	(3.43)	3.82	(2.49)	1.85	(3.36)	1.91	(0.29)
		EE_neg_55_R	-1.31	(1.38)	3.92	(1.85)	-1.26	(2.97)	3.85	(2.36)	1.15	(3.30)	1.94	(0.24)
		EE_neg_56_R	-0.31	(2.21)	3.54	(2.88)	-1.18	(3.06)	3.76	(2.67)	2.09	(3.43)	1.85	(0.36)

In study 1 (n = 13) participants evaluated each stimulus. In study 2 (n = 68) each participant evaluated only half of the dynamic stimuli, so each dynamic stimulus was evaluated by 34 participants. "_R" in the stimuli labels marks the reference stimuli with which the natural peak expression time was calculated.
