# Supplementary material for: Development and validation of a natural dynamic facial expression stimulus set
Source: PLoS One. 2023 Jun 28;18(6):e0287049. doi: 10.1371/journal.pone.0287049 (PMC10306207; doi:10.1371/journal.pone.0287049)
Supplement: S4 Table — The abbreviation “aP”in the video clip labels refers to the blinking starting after the peak expression (667ms). The abbreviation “bP” refers to the blinking starting before the peak expression. (PDF) [file pone.0287049.s004.pdf]

S4 Table. Mean Ratings and SDs of each Rating Dimension of Study 2 for Neutral Expression Stimuli

Elicitiation Method	Individual Clips	Study 2: Dynamic Stimuli Validation							
		Valence		Intensity		Genuineness		Perceived Edits	
		M	*(SD)*	*M*	*(SD)*	*M*	*(SD)*	*M*	*(SD)*
Posed									
	P_neu_aP_02	-0.32	(2.33)	3.15	(2.75)	2.91	(3.56)	1.91	(0.29)
	P_neu_aP_04	0.24	(2.84)	3.00	(2.76)	2.26	(3.68)	1.88	(0.33)
	P_neu_aP_06	0.29	(2.41)	2.82	(2.90)	2.94	(2.78)	1.94	(0.24)
	P_neu_aP_08	0.21	(2.43)	2.82	(2.54)	1.59	(3.64)	1.85	(0.36)
	P_neu_aP_10	0.12	(2.41)	2.94	(2.91)	2.21	(3.04)	1.76	(0.43)
	P_neu_aP_12	0.06	(2.40)	2.82	(2.44)	2.47	(3.73)	1.91	(0.29)
	P_neu_aP_14	1.21	(2.67)	3.00	(2.87)	2.44	(3.26)	1.88	(0.33)
	P_neu_aP_16	0.91	(2.75)	3.79	(2.77)	3.00	(2.55)	1.91	(0.29)
	P_neu_aP_18	-0.18	(2.76)	3.21	(2.89)	2.59	(3.46)	1.97	(0.17)
	P_neu_aP_20	-0.47	(2.67)	2.71	(2.29)	2.03	(3.57)	1.94	(0.24)
	P_neu_aP_24	-0.26	(2.43)	2.74	(2.77)	2.65	(3.41)	1.88	(0.33)
	P_neu_aP_26	0.09	(2.89)	3.12	(2.89)	2.21	(3.55)	1.91	(0.29)
	P_neu_aP_28	1.18	(2.94)	3.97	(2.94)	2.62	(3.16)	1.91	(0.29)
	P_neu_aP_30	-0.44	(2.19)	2.32	(2.40)	1.71	(2.99)	1.94	(0.24)
	P_neu_aP_32	-0.21	(2.71)	3.21	(2.77)	3.21	(3.20)	1.97	(0.17)
	P_neu_aP_34	0.12	(2.52)	2.29	(2.55)	2.03	(3.43)	1.85	(0.36)
	P_neu_aP_36	0.65	(2.60)	3.47	(2.57)	2.71	(3.48)	1.88	(0.33)
	P_neu_aP_38	0.32	(3.20)	3.09	(3.17)	2.59	(2.52)	1.88	(0.33)
	P_neu_aP_40	0.26	(2.59)	3.09	(2.71)	1.62	(3.52	1.85	(0.36)
	P_neu_aP_42	0.44	(2.81)	2.91	(2.87)	1.32	(4.43)	1.79	(0.41)
	P_neu_aP_44	0.44	(1.94)	2.68	(2.65)	2.41	(3.45)	1.82	(0.39)
	P_neu_aP_46	0.09	(2.81)	3.18	(2.69)	2.91	(3.07)	1.85	(0.36)
	P_neu_aP_50	0.29	(3.01)	3.15	(2.79)	2.00	(3.52)	1.85	(0.36)
	P_neu_aP_52	0.24	(3.08)	3.18	(2.78)	2.50	(3.37)	1.82	(0.39)
	P_neu_aP_54	0.15	(2.49)	2.85	(2.55)	1.91	(3.81)	1.88	(0.33)
	P_neu_bP_01	0.74	(2.55)	3.03	(2.67)	1.79	(3.36)	1.82	(0.39)
	P_neu_bP_03	0.00	(2.52)	2.65	(2.45)	1.68	(3.35)	1.91	(0.29)
	P_neu_bP_05	-0.41	(2.50)	2.56	(2.41)	2.65	(2.85)	1.88	(0.33)
	P_neu_bP_07	0.47	(2.36)	3.03	(2.67)	1.35	(3.58)	1.82	(0.39)
	P_neu_bP_09	-0.79	(2.80)	2.94	(2.47)	3.09	(2.87)	1.88	(0.33)
	P_neu_bP_11	-0.09	(2.93)	3.85	(2.57)	2.38	(3.19)	1.91	(0.29)
	P_neu_bP_13	0.29	(2.66)	3.35	(2.60)	1.41	(3.61)	1.91	(0.29)
	P_neu_bP_15	0.26	(2.42)	3.29	(2.60)	2.53	(3.63)	1.91	(0.29)
	P_neu_bP_17	0.03	(2.65)	3.06	(2.55)	2.91	(3.10)	1.88	(0.33)
	P_neu_bP_19	0.29	(2.08)	2.53	(2.49)	1.79	(3.47)	1.88	(0.33)
	P_neu_bP_21	0.24	(2.81)	3.38	(2.88)	2.26	(3.62)	1.97	(0.17)
	P_neu_bP_22	0.26	(2.57)	2.88	(2.76)	2.00	(3.70)	1.88	(0.33)
	P_neu_bP_23	1.18	(2.96)	3.56	(3.15)	3.62	(2.94)	2.00	(0.00)
	P_neu_bP_25	0.03	(2.80)	3.12	(2.72)	1.74	(4.14)	1.85	(0.36)
	P_neu_bP_27	0.09	(2.97)	2.97	(2.69)	2.41	(3.23)	1.85	(0.36)
	P_neu_bP_29	0.26	(2.59)	3.03	(2.78)	2.03	(4.01)	1.76	(0.43)
	P_neu_bP_31	-0.18	(2.15)	2.71	(2.59)	2.47	(3.82)	1.91	(0.29)
	P_neu_bP_33	-0.12	(2.18)	3.03	(2.59)	2.53	(3.03)	1.94	(0.24)
	P_neu_bP_35	0.24	(2.37)	2.62	(2.59)	1.79	(3.24)	1.94	(0.24)

Elicitiation Method	Individual Clips	Study 2: Dynamic Stimuli Validation							
		Valence		Intensity		Genuineness		Perceived Edits	
		M	*(SD)*	*M*	*(SD)*	*M*	*(SD)*	*M*	*(SD)*
Posed									
	P_neu_bP_37	0.26	(3.15)	3.71	(2.83)	1.74	(3.96)	1.94	(0.24)
	P_neu_bP_39	0.41	(2.13)	2.97	(2.72)	2.59	(2.83)	1.97	(0.17)
	P_neu_bP_41	-0.18	(2.48)	2.44	(2.50)	2.82	(3.38)	1.97	(0.17)
	P_neu_bP_43	0.12	(2.27)	2.29	(2.33)	2.21	(2.66)	1.91	(0.29)
	P_neu_bP_45	0.44	(2.67)	3.15	(2.43	2.18	(3.49)	1.91	(0.29)
	P_neu_bP_47	-0.47	(3.06)	2.82	(2.84)	2.88	(2.76)	1.88	(0.33)
	P_neu_bP_48	0.68	(2.84)	3.00	(2.75)	1.91	(3.53)	1.91	(0.29)
	P_neu_bP_49	0.18	(2.25)	2.74	(2.45)	2.21	(3.45)	1.85	(0.36)
	P_neu_bP_51	0.29	(2.48)	3.09	(2.50)	2.85	(3.28)	1.88	(0.33)
	P_neu_bP_53	-0.15	(3.02)	2.94	(2.80)	2.88	(3.01)	1.94	(0.24)
	P_neu_bP_55	0.26	(2.59)	2.47	(2.85)	1.68	(3.54)	1.88	(0.33)
	P_neu_bP_56	0.47	(3.19)	3.88	(3.24)	1.85	(4.10)	1.88	(0.33)
	P_neu_bP_57	-0.15	(2.97)	3.09	(3.02)	1.74	(3.70)	1.88	(0.33)
Event-Elicited									
	EE_neu_aP_02	-0.15	(3.11)	3.12	(2.56)	2.38	(3.77)	1.88	(0.33)
	EE_neu_aP_04	0.35	(2.36)	2.50	(2.74)	3.24	(2.83)	1.88	(0.33)
	EE_neu_aP_06	0.03	(2.83)	2.97	(2.52)	1.41	(3.12)	1.94	(0.24)
	EE_neu_aP_08	0.03	(1.80)	2.44	(2.44)	1.32	(3.53)	1.85	(0.36)
	EE_neu_aP_10	-0.53	(2.53)	2.32	(2.28)	2.53	(2.70)	1.85	(0.36)
	EE_neu_aP_12	1.21	(3.28)	3.85	(3.15)	0.71	(3.66)	1.82	(0.39)
	EE_neu_aP_14	-0.35	(2.56)	3.26	(2.49)	1.91	(3.30)	1.91	(0.29)
	EE_neu_aP_16	-0.47	(2.39)	2.32	(2.03)	1.79	(3.76)	1.88	(0.33)
	EE_neu_aP_20	1.56	(3.53)	4.29	(3.02)	2.76	(3.85)	1.85	(0.36)
	EE_neu_aP_24	0.06	(2.33)	2.82	(2.52)	1.41	(4.24)	1.88	(0.33)
	EE_neu_aP_26	0.53	(2.44)	2.74	(2.44)	2.03	(3.58)	1.82	(0.39)
	EE_neu_aP_28	0.68	(2.27)	2.68	(2.60)	1.38	(3.87)	1.82	(0.39)
	EE_neu_aP_30	0.68	(2.98)	3.65	(2.94)	2.24	(3.55)	1.79	(0.41)
	EE_neu_aP_32	0.35	(2.92)	3.41	(2.81)	2.44	(3.61)	1.94	(0.24)
	EE_neu_aP_34	0.09	(2.53)	2.74	(2.34)	3.12	(2.33)	1.88	(0.33)
	EE_neu_aP_36	0.59	(3.21)	3.71	(2.42)	1.88	(3.22)	1.82	(0.39)
	EE_neu_aP_38	-0.15	(2.51)	3.06	(2.32)	2.24	(3.27)	1.88	(0.33)
	EE_neu_aP_40	0.09	(2.66)	3.00	(2.57)	1.76	(3.23)	1.74	(0.45)
	EE_neu_aP_42	-0.32	(2.51)	2.91	(2.34)	1.50	(3.40)	1.79	(0.41)
	EE_neu_aP_44	-0.50	(2.72)	3.53	(2.98)	2.32	(3.07)	1.97	(0.17)
	EE_neu_aP_46	-0.62	(2.26)	2.74	(2.45)	2.79	(2.32)	1.88	(0.33)
	EE_neu_aP_50	-0.06	(2.89)	3.56	(2.67)	1.29	(3.43)	1.85	(0.36)
	EE_neu_aP_52	-0.71	(2.56)	2.41	(2.35)	2.62	(2.53)	2.00	(0.00)
	EE_neu_aP_54	1.00	(2.81)	2.94	(2.74)	1.74	(3.47)	1.79	(0.41)
	EE_neu_aP_56	0.09	(2.87)	3.03	(2.87)	2.47	(3.27)	1.91	(0.29)
	EE_neu_bP_01	-0.85	(2.97)	3.29	(2.60)	2.35	(3.50)	1.82	(0.39)
	EE_neu_bP_03	-0.15	(3.12)	3.79	(2.82)	1.35	(4.08)	1.79	(0.41)
	EE_neu_bP_05	-0.06	(2.74)	3.06	(2.73)	1.71	(3.57)	1.88	(0.33)
	EE_neu_bP_07	0.12	(2.65)	3.18	(2.59)	1.18	(3.97)	1.79	(0.41)
	EE_neu_bP_09	-0.15	(1.37)	1.62	(2.10)	2.88	(2.69)	1.88	(0.33)
	EE_neu_bP_11	0.00	(2.34)	3.15	(2.76)	1.85	(3.76)	1.94	(0.24)
	EE_neu_bP_13	1.06	(2.60)	2.91	(2.39)	1.88	(4.35)	1.85	(0.36)
	EE_neu_bP_17	-0.50	(2.62)	3.53	(2.45)	1.65	(3.15)	1.85	(0.36)
	EE_neu_bP_19	0.21	(2.53)	2.79	(2.28)	1.85	(3.50)	1.79	(0.41)
	EE_neu_bP_21	0.53	(2.82)	2.85	(2.68)	3.03	(3.26)	1.91	(0.29)

Elicitiation Method	Individual Clips	Study 2: Dynamic Stimuli Validation							
		Valence		Intensity		Genuineness		Perceived Edits	
		M	*(SD)*	*M*	*(SD)*	*M*	*(SD)*	*M*	*(SD)*
Event-Elicited									
	EE_neu_bP_22	-0.24	(2.64)	3.06	(2.52)	2.29	(3.57)	1.91	(0.29)
	EE_neu_bP_23	-0.15	(1.92)	2.56	(2.45)	1.65	(3.31)	1.85	(0.36)
	EE_neu_bP_25	-0.68	(2.64)	2.62	(2.73)	2.38	(3.19)	1.88	(0.33)
	EE_neu_bP_27	0.74	(3.33)	4.00	(2.77)	1.32	(3.66)	1.91	(0.29)
	EE_neu_bP_29	-0.50	(2.77)	2.76	(2.44)	2.21	(3.36)	1.82	(0.39)
	EE_neu_bP_31	0.56	(2.79)	3.21	(2.92)	1.50	(3.63)	1.91	(0.29)
	EE_neu_bP_33	-1.15	(2.68)	3.38	(2.49)	3.24	(2.69)	1.85	(0.36)
	EE_neu_bP_35	-0.12	(2.01)	2.41	(2.31)	3.06	(2.91)	1.88	(0.33)
	EE_neu_bP_37	0.76	(3.48)	3.56	(3.25)	2.88	(3.14)	1.91	(0.29)
	EE_neu_bP_39	0.41	(1.79)	2.15	(2.68)	2.21	(3.11)	1.91	(0.29)
	EE_neu_bP_41	0.21	(3.15)	3.24	(2.90)	3.62	(2.98)	1.94	(0.24)
	EE_neu_bP_43	0.03	(2.33)	2.62	(2.62)	2.71	(3.27)	1.85	(0.36)
	EE_neu_bP_45	-0.26	(2.70)	3.65	(2.73)	2.68	(3.57)	1.91	(0.29)
	EE_neu_bP_47	0.35	(2.98)	3.56	(2.78)	2.00	(3.60)	1.91	(0.29)
	EE_neu_bP_48	-0.32	(2.48)	2.76	(2.70)	2.18	(3.55)	1.97	(0.17)
	EE_neu_bP_49	0.18	(2.90)	3.41	(2.68)	1.97	(3.67)	1.97	(0.17)
	EE_neu_bP_51	-0.38	(2.05)	2.74	(2.71)	2.09	(3.39)	1.79	(0.41)
	EE_neu_bP_53	0.32	(2.37)	2.91	(3.18)	2.85	(4.14)	1.94	(0.24)
	EE_neu_bP_55	0.32	(2.84)	3.44	(2.70)	2.18	(3.26)	1.88	(0.33)
	EE_neu_bP_57	0.15	(2.31)	2.71	(2.60)	2.53	(3.12)	1.91	(0.29)

The abbreviation “aP“ in the video clip labels refers to the blinking starting after the peak expression (667ms). The abbreviation “bP” refers to the blinking starting before the peak expression.
