# Supplementary material for: Development and validation of a natural dynamic facial expression stimulus set
Source: PLoS One. 2023 Jun 28;18(6):e0287049. doi: 10.1371/journal.pone.0287049 (PMC10306207; doi:10.1371/journal.pone.0287049)
Supplement: S1 File — (PDF) [file pone.0287049.s005.pdf]

S1 File. Questions Regarding the Success of the Elicitation Conditions during the Video Recording.

After the video recording session, the model was asked five open questions which she could answer in a written format.

These five questions were:

1. Could you imagine the described situations well?

2.

   a) Could you imagine the situations for the whole duration of each long video?

   b) For how long could you imagine the described situations?

3. Did you think about anything during the posed videos?

4. Do you want to add anything else?
