# Supplementary material for: Development and validation of a natural dynamic facial expression stimulus set
Source: PLoS One. 2023 Jun 28;18(6):e0287049. doi: 10.1371/journal.pone.0287049 (PMC10306207; doi:10.1371/journal.pone.0287049)
Supplement: S2 File — (PDF) [file pone.0287049.s006.pdf]

S2 File. German Explanations of the Experimental Flow and the Rating Scales

Ihre Aufgabe innerhalb der Studie

Gleich beginnt die Studie, weshalb hier noch einige Informationen bereitgestellt werden.

Ihre Aufgabe ist es, sich kurze Video-Clips anzuschauen und anschließend zu bewerten.
Diese Video-Clips zeigen verschiedene emotionale Gesichtsausdrücke.
Da die Video-Clips nur ca. eine Sekunde lang sind, seien Sie bitte aufmerksam.
Nach jedem Video-Clip werden vier Fragen eingeblendet, diese sind:

1. Wie würden Sie den Gesichtsausdruck in dem letzten Video-Clip beschreiben?

Diese Frage bezieht sich allgemein auf die Dimension positiv bis negativ, d.h. Sie bewerten, ob ein Gesichtsausdruck (eher) positiv, neutral oder (eher) negativ wirkt.

2. Wie intensiv würden Sie den Gesichtsausdruck in dem letzten Video-Clip beschreiben?

Intensiv bezieht sich darauf wie stark der Gesichtsausdruck ausgeprägt ist.

3. Wie authentisch würden Sie den Gesichtsausdruck des letzten Video-Clips beschreiben?

Authentisch bezieht sich auf die Echtheit/Glaubwürdigkeit des Gesichtsausdrucks, d.h. ob Sie glauben, dass der Gesichtsaudruck nicht gespielt ist.

4. Glauben Sie der Video-Clip wurde nachbearbeitet?

Nachbearbeitet bedeutet dass der Video-Clip nach der Aufnahme verändert wurde, indem z.B. kleine Sequenzen entfernt oder hinzugefügt wurden. Keine Sorge, falls Sie nur bei wenigen Video-Clips glauben, dass Sie bearbeitet wurden.

Sie können einfach einen Wert auf den eingeblendeten Skalen bzw. bei der 4. Frage "Ja" oder "Nein" anklicken. Nach der vierten Frage, kommen Sie durch Drücken des Weiter-Buttons automatisch zum nächsten Video-Clip.

Nach jedem Viertel der Video-Clips wird eine Pause eingelegt.

Wenn Sie bereit sind, können Sie mit dem Weiter-Button in eine kurze Testrunde starten.
Um einen Video-Clip zu starten, klicken Sie diesen bitte an.
Das Laden der Video-Clips kann unter Umständen etwas Zeit benötigen, bitte seien Sie geduldig.

Falls, nach einer kurzen Wartezeit, ein Video-Clip nicht lädt, können Sie die Seite auch neu laden, ohne dass ihre vorherigen Daten verloren gehen.
