# Supplementary material for: Development and validation of a natural dynamic facial expression stimulus set
Source: PLoS One. 2023 Jun 28;18(6):e0287049. doi: 10.1371/journal.pone.0287049 (PMC10306207; doi:10.1371/journal.pone.0287049)
Supplement: S3 File — (PDF) [file pone.0287049.s007.pdf]

S3 File. English Explanations of the Experimental Flow and the Rating Scales.

Your task in the study

The study is about to begin, which is why some important information is provided here.

Your task is to watch short video clips and then rate them. The video clips show different emotional facial expressions. As the video clips are only one second long, please pay attention.

Four questions will appear after each video clip:

1. How would you describe the facial expression in the last video clip?

This question generally relates to the dimension positive to negative, i.e. you rate whether a facial expression is (rather) positive, neutral or (rather) negative.

2. How intensely would you describe the facial expression in the last video clip?

Intense refers to how strong the facial expression is.

3. How genuine would you describe the facial expression of the last video clip?

Genuine refers to the authenticity/believability of the facial expression, i.e. whether you believe the facial expression is or is not fake.

4. Do you think the last video clip was edited?

Edited refers to the video clip having been modified after recording, e.g. by removing or adding small sequences. Do not worry if you think only a few video clips have been edited.

You can simply click on a value on the displayed scales or in case of the 4th question "Yes" or "No". After the fourth question, pressing the “Next” button will automatically take you to the next video clip.

There will be a break after each quarter of all video clips.

When you are ready, you can start a short test round by clicking on the “Next” button. To start a video clip, please click on it. The video clips may take some time to load, please be patient. If after a short waiting period, a video clip does not load, you can also reload the page without losing your previous data.
