# Supplementary material for: Development and validation of a natural dynamic facial expression stimulus set
Source: PLoS One. 2023 Jun 28;18(6):e0287049. doi: 10.1371/journal.pone.0287049 (PMC10306207; doi:10.1371/journal.pone.0287049)
Supplement: S4 File — (PDF) [file pone.0287049.s008.pdf]

S4 File. Assumption Tests for the Used Statistical Analyses.

Assumption Tests for the Exploratory Analyses on Potential Differences between the First Onset Frames of Positive and Negative Stimuli (Study 1)

Valence ratings were not normally distributed, neither for positive expressions (W = 0.97, $p < .001$), nor for negative expressions (W = 0.94, $p < .001$), as Q-Q-plots and Shapiro-Wilk tests revealed. Normal distribution could once again not be assumed as Q-Q-plots and Shapiro-Wilk test showed for the *valence* ratings of posed (W = 0.95, $p < .001$) and event-elicited positive expressions (W = 0.97, $p < .001$). Also, for the *valence* ratings of negative expression stimuli normal distribution could not be assumed, as Shapiro-Wilk tests were significant for posed (W = 0.93, $p < .001$) and event-elicited expressions (W = 0.95, $p < .001$).

Intensity ratings did not follow a normal distribution, neither for positive (W = 0.97. $p <$ .001) or negative expressions (W = 0.97, $p < .001$) overall nor when divided further into posed ($W_{positive}$ = 0.97, $p_{positive} < .001$; $W_{negative}$ = 0.97. $p_{negative} < .001$) and event-elicited ($W_{positive}$ = 0.97. $p_{positive} < .001$; $W_{negative}$ = 0.97. $p_{negative} < .001$), as Q-Q-plots and Shapiro-Wilk test revealed.

Assumption Tests for the Hypotheses Testing of Study 2

To test hypothesis 1, the *valence* ratings of negative expression stimuli were explored. On the basis of central limit theorem normal distribution was assumed for the *valence* ratings of negative expression stimuli (M = -0.47, SD = 3.06) in both stimuli sets. As Levene's test was not significant, $F(1, 3398) = 2.34$, $p = .13$, homogeneity of variances could be assumed for the *valence* ratings of negative expression stimuli in both stimuli sets. Therefore, a one-way ANOVA was conducted to explore possible differences between the to stimuli sets.

For hypothesis 2 the same assumption tests were computed. For video clips with a positive expression, normal distribution was again assumed based on central limit theorem, and

Levene's test revealed homogeneity of variances of *valence* ratings once more, $F(1, 2786) = 0.24$, $p = .62$. Therefore a one-way ANOVA was conducted to compare the two stimuli sets

For hypothesis 3 the *valence* ratings of neutral expression stimuli were investigated. Q-Q-plots and Shapiro-Wilk test indicated no normal distribution for *valence* ratings of neutral expression stimuli, $W = 0.909$, $p < .001$. Furthermore, Levene's tests were non-significant for stimuli sets and indicated no homogeneity of variances between the two stimuli sets, $F(1, 9994) = 2.29$, $p = .13$, but was highly significant for expressions, $F(2, 9993) = 160.1$, $p < .001$. Kruskal-Wallis test was conducted since the standard deviations of *valence* ratings for each group were similar.

For hypothesis 4 the standard deviations of *genuineness* ratings of the stimuli were explored. Shapiro-Wilk test, Q-Q-plots and histograms all revealed a normal distribution of the standard deviations *genuineness* ratings for all expressions and both stimuli sets. Levene's test revealed homogeneity of variances between the groups, $F(5, 198) = 0.37$, $p = .87$.

For hypothesis 5 the *intensity* ratings of negative and positive expression stimuli were explored. Normal distribution was assumed based on central limit theorem. Levene's test did not reveal homogeneity of variances $F(3, 6184) = 8.03$, $p < .001$. As the standard deviations were similar, a Kruskal-Wallis test was computed.

For hypothesis 6 the mean *genuineness* ratings of low- and high-intensity stimuli were explored. Shapiro-Wilk tests and Q-Q-plots revealed normal distribution for mean *genuineness* ratings per stimulus, but Levene's test showed no homogeneity in variances, $F(3, 290) = 5.93$, $p < .001$. Consequently, Kruskal-Wallis tests were conducted. For the exploratory analyses of hypothesis 6, the neutral expression stimuli were excluded from the data. Normal distribution was again assumed based on central limit theorem, however Levene's test did not reveal homoscedasticity, $F(3, 178) = 5.72$, $p < .001$.

For hypothesis 7 the *genuineness* ratings of posed and event-elicited positive expression stimuli were explored. Normal distribution was again assumed on the basis of central limit theorem. Homogeneity of variances could not be assumed due to a significant result of Levene's test, $F(3, 2784) = 3.30$, $p = .02$.

For hypothesis 8 the *genuineness* ratings of posed and event-elicited negative expression stimuli were explored. Normal distribution was assumed based on central limited theorem, and Levene's test revealed homogeneity of variances, $F(3, 3396) = 2.53$, $p = 0.06$. Consequently, a mixed ANOVA was conducted to compare the two stimuli sets.

For hypothesis 9 the *genuineness* ratings of posed and event-elicited neutral expression stimuli were explored. Q-Q-plots, histograms as well as Shapiro-Wilk tests showed no normal distribution. Levene's test showed no homogeneity of variances, $F(3, 3804) = 5.19$, $p = .001$.
