# Supplementary material for: Development and validation of a natural dynamic facial expression stimulus set
Source: PLoS One. 2023 Jun 28;18(6):e0287049. doi: 10.1371/journal.pone.0287049 (PMC10306207; doi:10.1371/journal.pone.0287049)
Supplement: S5 File — (PDF) [file pone.0287049.s009.pdf]

S5 File. Results – Study 1: First Onset Frame Validation.

Descriptive Statistics

In study 1, the participants rated the first onset frame of the developed stimuli on three rating scales, namely valence, certainty and intensity. Unfortunately, participants skipped the certainty rating question for 6.01% of all onset frames, so the results could not be analysed. For positive expression stimuli the mean *valence* rating of the first onset frame was 2.27 (*SD* = 2.16), while it was -1.63 (*SD* = 1.86) for negative expression stimuli. For positive expression stimuli the mean *intensity* rating of the first onset frame was *M* = 4.63 (*SD* = 2.01), and for negative expression stimuli it was *M* = 3.93 (*SD* = 2.05). *Mood* overall declined slightly during the survey with a mean rating of 3.39 (*SD* = 0.96) before the stimuli rating task and 3.31 (*SD* = 0.63) after.

Next to the mean *valence* rating, the minimum and maximum *valence* ratings for the intended as positive and intended as negative stimuli were investigated. Some stimuli that were intended as positive received a *valence* rating below zero (see S5 Figure), while some stimuli that were intended as negative received a *valence* rating above zero (see S5 Figure). When checking the ratings for outliers, only one extreme outlier rating was found for *valence*, but none for *intensity*. The rating was excluded from all statistical analyses, whereas the stimulus was reviewed, but not excluded from the actual stimulus set, since it was only one rating of that stimulus, which could have been made by accident. It was further investigated how many participants rated stimuli in the opposite direction as intended. Especially, one participant rated the *valence* of intended as positive stimuli remarkably often below zero, while another rated negative stimuli remarkably often above zero. The stimuli that received a valence rating opposite to the intended *valence* were checked by the experimenters. If multiple participants rated the *valence* opposite to the expected *valence* and the experimenters excluded participant bias as an alternative explanation, the stimulus was excluded from the stimulus set before the

second validation study took place. Otherwise, the stimulus was not excluded, but the ratings within study 2 were checked in detail. In total, four positive expression stimuli (P_pos_20_R, P_pos_56, EE_pos_27, EE_pos_47) and two negative expression stimuli (EE_neg_03, EE_neg_07) were excluded from the stimulus set and study 2. The mean ratings of study 2 of the remaining stimuli which had received a rating opposite to the intended *valence* in study 1, can be seen in the supplementary material (S5 Table).

S5 Figure. Valence Ratings of First Onset Frames of Positive Expression Stimuli (Upper Panel) and Negative Expression Stimuli (Lower Panel).

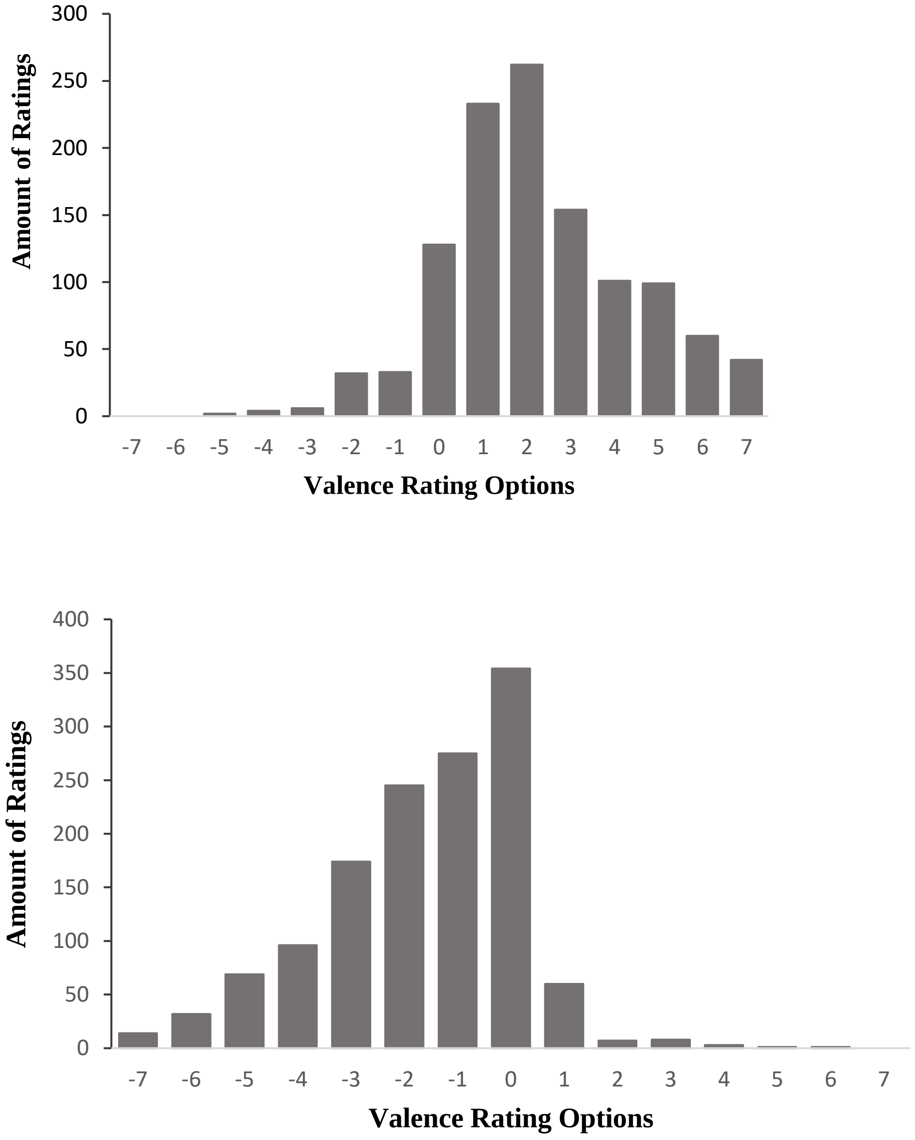

S5 Table. Mean Ratings and *SD*s of Study 2 of the Dynamic Stimuli with Doubtful Onset Frames

Expression	Elicitation	Stimulus	Dynamic Stimulus Study					
			Valence		Intensity		Genuineness	
			M	*(SD)*	*M*	*(SD)*	*M*	*(SD)*
Positive	Posed	P_pos_43_R	0.88	(3.34)	3.56	(3.02)	3.15	(3.38)
	Event-Elicited	EE_pos_38	0.24	(3.04)	3.56	(3.14)	1.71	(3.99)
	Event-Elicited	EE_pos_41	0.76	(3.37)	4.32	(2.92)	2.09	(4.01)
Negative	Posed	P_neg_19_R	-0.41	(2.84)	3.85	(2.83)	1.26	(3.89)
	Posed	P_neg _31_R	-0.09	(2.81)	4.35	(2.93)	2.50	(3.33)
	Posed	P_neg_39_R	-0.38	(2.77)	3.41	(2.51)	1.68	(3.24)
	Event-Elicited	EE_neg_10_R	-0.18	(3.21)	3.91	(2.64)	1.79	(3.29)
	Event-Elicited	EE_neg _18_R	-0.97	(2.85)	3.76	(2.15)	2.32	(3.07)
	Event-Elicited	EE_neg _32	-0.29	(2.83)	3.35	(2.50)	2.03	(3.41)
	Event-Elicited	EE_neg _43_R	-0.03	(2.69)	2.37	(2.53)	2.44	(3.08)

These stimuli have not received a mean valence rating of the first onset frames that was opposite as intended, mostly they received only one valence rating which was opposite to the intended valence in study 1 (*n* = 13).

Exploratory Analyses of Potential Differences between the First Onset Frames of Positive and Negative Stimuli

It was investigated whether the *valence* ratings of positive and negative expression onset frames differed from each other. Due to different amounts of positive and negative expression stimuli no paired sample tests could be used, but instead independent sample tests had to be employed. Non-parametric test were indicated, therefore a Mann-Whitney U test was computed which revealed a significant difference between the *valence* of positive (*Mdn* = 2.00) and negative (*Mdn* = -1.00) first onset frames, U ($N_{positive}$ = 1156, $N_{negative}$ = 1339) = 109722, Z = -37.28, p < .001, r = 0.75.

A one-sample Wilcoxon signed rank test indicated that that positive expression onset

frames differed significantly from a neutral *valence* rating, $p < .001$, $r = 0.74$, and that the negative expression onset frames received *valence* ratings significantly below zero (i.e., neutral), $p < .001$, $r = 0.68$.

Moreover, the *valence* ratings of posed and the event-elicited onset frames were compared for each expression. A Mann-Whitney U test revealed that posed positive (Mdn = 2.00) and event-elicited positive expressions (Mdn = 2.00) differed significantly, $U(n_{posed} = 520, n_{event\text{-}elicited} = 636) = 183909$, $Z = 3.33$, $p < .001$, $r = 0.10$, with event-elicited positive expressions receiving higher *valence* ratings than posed ones, $U(n_{posed} = 520, n_{event\text{-}elicited} = 636) = 183909$, $Z = 3.33$, $p < .001$, $r = 0.10$. Posed negative (Mdn = -1.00) and event-elicited negative expressions (Mdn = -2.00) differed significantly as a Mann-Whitney U test showed, $U(n_{posed} = 728, n_{event\text{-}elicited} = 611) = 201830$, $p = .003$, $r = 0.08$. *Therefore, for both positive and negative expressions, the event-elicited expressions received more extreme valence ratings than the posed expressions.*

Comparing the *intensity* ratings of the different expressions and elicitation methods was also of interest. A Mann-Whitney U test indicated a highly significant difference in *intensity* between positive (Mdn = 5.00) and negative (Mdn = 4.00) expression onset frames, $U(N_{positive} = 1156, N_{negative} = 1339) = 626192$, $Z = -8.32$, $p < .001$, $r = 0.17$. Further, a Mann-Whitney U test revealed a highly significant difference in *intensity* of posed (Mdn = 5.00) and event-elicited positive expressions (Mdn = 5.00), $U(n_{posed} = 520, n_{event\text{-}elicited} = 636) = 184559$, $Z = 3.44$, $p < .001$, $r = 0.10$. A one-sided Mann-Whitney U test revealed that event-elicited positive expressions were perceived as more intense than posed positive expressions, $U(n_{posed} = 520, n_{event\text{-}elicited} = 636) = 184559$, $Z = 3.44$, $p < .001$, $r = 0.10$. A significant difference between posed (Mdn = 4.00) and event-elicited expressions (Mdn = 4.00) could also be found for negative expressions by computing a Mann-Whitney U test, $U(n_{posed} = 728, n_{event\text{-}elicited} = 611) = 240310$, $Z = 2.57$, $p = .010$, $r = 0.07$. One-sided testing revealed once more, that event-elicited

expressions were rated as more intense than posed expressions, $U(n_{posed}$= 728, $n_{event\text{-}elicited}$ = 611) = 240310, Z = 2.57, p = .005, r = 0.07. *To conclude, the first onset frames of positive expression stimuli received higher intensity ratings than those of negative expression stimuli, and the first onset frames of event-elicited stimuli were perceived as more intense than those of posed stimuli, for both positive and negative expression stimuli.*
